# Comparative genomic profiling of transport inhibitor Response1/Auxin signaling F-box (TIR1/AFB) genes in eight *Pyrus* genomes revealed the intraspecies diversity and stress responsiveness patterns

**DOI:** 10.3389/fgene.2024.1393487

**Published:** 2024-05-10

**Authors:** Sheng Yang, Xiaomei Yu, Xinke Gao, Kinza Fatima, Muhammad Tahir Ul Qamar

**Affiliations:** ^1^ Pomology Institute, Shanxi Agricultural University, Shanxi Key Laboratory of Germplasm Improvement and Utilization in Pomology, Taiyuan, Shanxi, China; ^2^ College of Horticulture, Shanxi Agricultural University, Jinzhong, Shanxi, China; ^3^ Integrative Omics and Molecular Modeling Laboratory, Department of Bioinformatics and Biotechnology, Government College University Faisalabad (GCUF), Faisalabad, Pakistan

**Keywords:** *Pyrus*, pangenome-wide, TIR1/AFBs, evolutionary pattern, gene ontology, fruit hardening disease, abiotic stress, drought stress

## Abstract

In the genomics of plants and the phytoecosystem, Pyrus (pear) is among the most nutritious fruits and contains fiber that has great health benefits to humans. It is mostly cultivated in temperate regions and is one of the most cultivated pome fruits globally. Pears are highly subjected to biotic and abiotic stresses that affect their yield. TIR1/AFB proteins act as auxin co-receptors during the signaling of nuclear auxins and play a primary role in development-related regulatory processes and responses to biotic and abiotic stresses. However, this gene family and its members have not been explored in *Pyrus* genomes, and understanding these genes will help obtain useful insights into stress tolerance and ultimately help maintain a high yield of pears. This study reports a pangenome-wide investigation of TIR1/AFB genes from eight *Pyrus* genomes: Cuiguan (*Pyrus pyrifolia*), Shanxi Duli (*P. betulifolia*), Zhongai 1 [(*P. ussuriensis × communis*) × spp.], Nijisseiki (*P*. *pyrifolia*), Yunhong No.1 (*P*. *pyrifolia*), d’Anjou (*P. communis*), Bartlett v2.0 (*P. communis*), and Dangshansuli v.1.1 (*P. bretschneideri*). These genes were randomly distributed on 17 chromosomes in each genome. Based on phylogenetics, the identified TIR1/AFB genes were divided into six groups. Their gene structure and motif pattern showed the intraspecific structural conservation as well as evolutionary patterns of *Pyrus* TIR1/AFBs. The expansion of this gene family in *Pyrus* is mainly caused by segmental duplication; however, a few genes showed tandem duplication. Moreover, positive and negative selection pressure equally directed the gene’s duplication process. The GO and PPI analysis showed that *Pyrus TIR1/AFB* genes are associated with abiotic stress- and development-related signaling pathways. The promoter regions of *Pyrus TIR1/AFB* genes were enriched in hormone-, light-, development-, and stress-related *cis* elements. Furthermore, publicly available RNA-seq data analysis showed that *DaTIR1/AFBs* have varied levels of expression in various tissues and developmental stages, fruit hardening disease conditions, and drought stress conditions. This indicated that *DaTIR1/AFB* genes might play critical roles in response to biotic and abiotic stresses. The DaTIR1/AFBs have similar protein structures, which show that they are involved in the same function. Hence, this study will broaden our knowledge of the TIR1/AFB gene family in *Pyrus*, elucidating their contribution to conferring resistance against various environmental stresses, and will also provide valuable insights for future researchers.

## Introduction

Indole-3-acetic acid (IAA) is the most common and first auxin to be discovered in plants. The auxin hormone is widely involved in plant growth, development, and morphogenesis (Kepinski and Leyser, 2005a; Zhao, 2010). The functions of auxins in plants are mainly controlled by modulating auxin synthesis metabolism, signal transduction (Petra et al., 2009), and polar transport (Mockaitis and Estelle, 2008). The classical pathway that mediates auxin signaling is known as transport inhibitor response1/auxin signaling F-box (*TIR1/AFB*)-mediated auxin regulation mechanism. The first auxin receptor protein to be reported was TIR1. Later, AFBs were also identified as the members of this family. The TIR1 genes belong to the F-box family of proteins whose members have highly conserved N-terminus F-box and C-terminus leucine-rich repeat (LRR) domains. The F-box domain of this family comprises the important component of the E3 ubiquitin ligase complex, which is involved in the degradation of auxin/indoleacetic acid (AUX/IAA) proteins (Hu et al., 2012).

AUX/IAA is one of the key gene families that are involved in quick response to fluctuations in auxin concentration. These gene family members are activated when the auxin level declines, form a dimer by binding to the auxin response factor (ARF), and regulate the auxin-induced gene expression. When auxin levels are high, the AUX/IAA proteins bind to the TIR1/AFBs, thus becoming degraded through ubiquitination that eliminates the inhibitory effect of AUX/IAAs. Auxin plays a major role in the growth, development, and morphogenesis of plants. Auxin response and signal transduction are strongly associated with the TIR1/AFB and AUX/IAA proteins (Paponov et al., 2008; Su et al., 2023a). Thus, the TIR1/AFB-AUX/IAA pathway is involved in the plant’s mechanism of the auxin signal’s perception, transduction, and response. It has also been demonstrated that auxin binding to TIR1/AFBs requires partial involvement of AUX/IAAs (Calderón Villalobos et al., 2012). *Arabidopsis thaliana* contains six *TIR1/AFBs* as well as 29 *AUX/IAAs* that are involved in diverse functions of auxin in this plant (Salehin et al., 2015).

Certain growth-related processes primarily regulated by auxin, including the elongation of the hypocotyl and lateral root development, are impaired in the mutants of tir1, having their functions lost (Ruegger et al., 1998). Overexpression of *AtTIR1* leads to primary root growth inhibition, formation of a lateral root, and agravitropic root tips (Gray et al., 1999). *TIR1* is the substrate receptor for the SKP-Cullin (SCF) E3 complex, which uses the ubiquitin-26S proteasome machinery to specifically break down Aux/IAA proteins; thus, SCF plays important roles in regulating cellular processes, including signal transduction (Xie et al., 2019). By directly binding to SCFTIR1, auxin molecules have the potential to enhance the interaction between the Aux/IAA and SCFTIR1 complex; *TIR1* has been identified as an auxin receptor (Kepinski and Leyser, 2005b; Dharmasiri et al., 2005). Shortly after the identification of *TIR1*, the three genes with the names *AFB1*, *AFB2*, and *AFB3* were discovered; these genes function similarly to *TIR1* (Tahir ul Qamar et al., 2020). It is important to note that all four genes—*TFR1*, *AFB1*, *AFB2m*, and *AFB3*—have involvement in the way plants respond to auxin in the root, but *AFB1* does not form an SCF complex as effectively as *TIR1*, *AFB2*, and *AFB3* (Yu et al., 2015). Both *AFB4* and *AFB5* have been shown to have auxin receptor functions. The *afb4* loss-of-function mutant seedlings show no discernible growth defects (Prigge et al., 2016).

Overexpression of *AtTIR1* in *A. thaliana* led to the overproduction of lateral roots (Chen et al., 2011). Moreover, the *AtAFB3* gene played a significant role in the lateral root development in response to nitrate treatment (Vidal et al., 2010). In the *afb4* loss-of-function mutant seedlings, the number of lateral roots developed is lower than in the wild-type seedlings, suggesting that *AFB4* is also involved in the development of lateral roots in *Arabidopsis.* The *afb4* mutant also showed other defects in development-related processes, such as delayed flowering, lower height, and hypocotyls (Hu et al., 2012). ABA treatment generally inhibits the development of lateral roots in *Arabidopsis;* however, the overexpression of *AtAFB2* has been demonstrated to counteract this inhibition, suggesting a response pathway from abiotic stress to this specific gene (Chen et al., 2012). Furthermore, the *Arabidopsis TIR1* and *AFB2* are required to inhibit the lateral root development by ABA or osmotic stress under drought stress (Chen et al., 2012). In rice, *TIR1* and *AFB2* were significantly down-expressed in spikelets under drought stress (Sharma et al., 2018). In maize, tomato, and potato crops, the expression of *TIR1* was increased in seedlings when exposed to drought stress (Benny et al., 2019). The knockdown of soybean *GmTIR1* and *GmAFB3* genes resulted in fewer nodule numbers (Cai et al., 2017). Pears are also affected by fruit hardening disorder in which the top region of the pear is hardened, thus affecting the fruit quality and its commercial value. Because the pear is among the most highly cultivated and commercially important fruits, this disease and its related mechanism have been investigated in several studies (Liu et al., 2021).

Pears are one of the most significant temperate fruit trees globally and belong to the Rosaceae family and the *Amygdaloideae* subfamily. Pears have been cultivated for over 3,000 years, with 39 billion tons being delivered worldwide yearly (Wu et al., 2014; Ferradini et al., 2017; Wu et al., 2018). Presently, 22 species of pear with 5000 accessions have been reported. These five species are majorly cultivated for fruit production, including *Pyrus* bretschneideri, *P*. *pyrifolia*, *P. communis*, *P. ussuriensis,* and *P. sinkiangensis* (Li et al., 2022). Most cultivated pears have a diploid genome (2n = 34) that is extremely heterozygous and has several repeating sequences (Chen et al., 2023). Pear is an economical fruit with a sweet taste and great nutritional value. Abiotic stresses such as drought and biotic stresses limit the cultivation area, affecting their growth and yield. Therefore, there is an urgent need to address this situation.

TIR1/AFB genes have been shown to have important regulatory roles in auxin-regulated responses, making these major regulators of plant growth and development that are often halted by environmental factors. In this project, TIR1/AFB gene family members have been identified from eight pear genomes: Cuiguan, Shanxi Duli, Zhongai 1, Nijisseiki, Yunhong No.1, d’Anjou, Bartlett v2.0, and Dangshansuli’ v.1.1 (Chen et al., 2023). Characterization and phylogenetic analysis were performed to determine the intraspecific evolutionary relationships among these genes. The results of the analysis of protein profiles and intron/exon structures supported the classification of the *Pyrus TIR1/AFB* genes. In addition, the expression profiles of *Pyrus TIR1/AFB* genes showed that these genes had different expression patterns under abiotic (drought) and biotic stresses (disease conditions). The results of this study might help better understand the role of *Pyrus TIR1/AFBs* in the biotic as well as abiotic stress response of pears and provide a foundation for identifying candidate genes involved in drought response and fruit hardening disease.

## Materials and methods

### Identification and physiochemical characterization of *Pyrus* TIR1/AFBs

The six *A. thaliana* TIR1/AFB protein sequences were retrieved using the Arabidopsis Information Resource website (https://www.arabidopsis.org/). The protein sequence FASTA files of Cuiguan, Shanxi Duli, Zhongai 1, Nijisseiki, Yunhong No.1, d’Anjou, Bartlett v2.0, and Dangshansuli’ v.1.1 were used as subject sequences to run the BLAST+ command-line tool. First, local databases for the eight Pyrus proteomes were created, and then the BLASTp search was conducted against each Pyrus proteome sequence database using TIR1/AFB protein sequences as the queries. Then, the resulting BLAST hits were filtered by steps including isoforms and duplicate removal.

The candidate *Pyrus* TIR1/AFB sequences were searched for the F-box protein family domains using the NCBI conserved domain database (CDD) (https://www.ncbi.nlm.nih.gov/Structure/cdd/wrpsb.cgi) (Marchler-Bauer et al., 2011) and the InterPro (https://www.ebi.ac.uk/interpro/) (Hunter et al., 2009) database to identify the final protein family sequences. Physicochemical characteristics, including molecular weight, their isoelectric point (pI), aliphatic index (AI), instability index II), and the grand average of hydropathicity (GRAVY) values were predicted by using the ExPASy ProtParam tool (https://web.expasy.org/protparam/) (Gasteiger et al., 2005). The subcellular localization of these TIR1/AFB proteins was predicted by using the WoLF PSORT tool (https://wolfpsort.hgc.jp/) (Horton et al., 2007).

### Phylogenetic analysis, conserved motifs, and gene structure analysis of *Pyrus* TIR1/AFBs

Phylogenetic analysis was conducted to evaluate the intraspecific evolutionary links among *Pyrus* TIR1/AFBs. A multiple sequence alignment of seven Cuiguan, six Shanxi Duli, six Zhongai 1, seven Nijisseiki, six Yunhong No.1, nine d’Anjou, five Bartlett v2.0, four Dangshansuli’ v.1.1, 6 *A. thaliana* (Kepinski and Leyser, 2005b), 18 *Brassica juncea* (Cai et al., 2000), and eight *Populus* (Shu et al., 2015) protein sequences was performed using ClustalW (Thompson et al., 2003). A phylogenetic tree was constructed using the IQTREE Web Server (http://iqtree.cibiv.univie.ac.at/) (Trifinopoulos et al., 2016) using the maximum likelihood (ML) method performing model selection *via* ModelFinder and bootstrap replicates of 1000. The tree was visualized and edited using the online Interactive Tree of Life server, iTOL (https://itol.embl.de/) (Letunic and Bork, 2007).

To evaluate the conserved common motifs present in all eight Pyrus TIR1/AFB sequences, the multiple Expectation Maximization for Motif Elicitation (MEME, https://meme-suite.org/meme/) (Bailey et al., 2009) tool was used. The conserved motif number was set to 20 to search for each sequence. The gene structures were constructed by using CDS and genomic sequences through the Gene Structure Display Server (GSDS 2.0, https://gsds.gao-lab.org/) (Hu et al., 2015). The identified motifs and gene structures were visualized using TBtools (Chen et al., 2018).

### Chromosomal distribution, Ka/Ks rate, and gene duplication analysis

The chromosomal positions for each Pyrus *TIR1/AFB* were retrieved from the GFF/GFF3 files and mapped to chromosomes by using the gene location visualization tool of the TBtools software (Chen et al., 2018). Depending on whether the shorter gene’s length covered 70% of the longer gene and whether the two aligned genes’ similarity was equal to or greater than 70%, *TIR1/AFB* gene duplication occurrences were identified (Tahir ul Qamar et al., 2023). The genes were also checked to determine whether the duplication pattern was segmental or tandem. To predict the selection pressure for the duplicated genes, Ka/Ks values were also predicted using DnaSP v.6 software (Le et al., 2011; Rozas et al., 2017). Depending on whether the Ka/Ks ratio was more than, equal to, or less than one, purifying, neutral, or positive selection was analyzed (Zia et al., 2022). Furthermore, the divergence time for the duplicated gene pairs was also calculated by using the formula t = Ks/2λ×10^−6^, where the λ value for dicots, 1.5 × 10^−8^, calculates the time of duplication in million-year units (Fatima et al., 2023a).

### PPI and GO enrichment analysis

The STRING database (von Mering et al., 2003) was used to analyze the PPIs among the Pyrus TIR1/AFB proteins using the amino acid sequences. The top 10 interactions were set to perform the prediction, and the interactions threshold was kept at 0.4. Furthermore, the PPI network was visualized by using Cytoscape software (Shannon et al., 2003). The GO enrichment analysis components, biological processes (BPs), cellular components (CCs), and molecular functions (MFs) were predicted using the PANNZER database (pannzer2 (helsinki.fi)) (Törönen et al., 2018).

### *Cis*-regulatory elements prediction and expression profiling of *Pyrus TIR1/AFBs*

To predict the *cis*-regulatory elements, the 2-kb sequences upstream of the translation start site of *Pyrus TIR1/AFB* genes were retrieved and used to search the PlantCARE online tool (http://bioinformatics.psb.ugent.be/webtools/plantcare/html/) (Rombauts et al., 1999).

To gain insights into the expression pattern of *Pyrus* Dangshansuli *TIR1/AFBs*, transcriptomic RNA-seq data of different developmental stages of pear fruit (BioProject: PRJNA309745), under drought stress (BioProject: PRJNA655255), and fruit hardening disease (BioProject: PRJNA763913) were obtained from the SRA-NCBI database (https://www.ncbi.nlm.nih.gov/sra). The Dangshansuli genome and annotation (GFF) files were downloaded from the Pear Genomics Database (PGDB) (http://pyrusgdb.sdau.edu.cn/) (Chen et al., 2023). The quality of the reads was evaluated by using the FastQC tool (Wingett and Andrews, 2018). The indexes of the *Pyrus* genome used were built by using HISAT (Kim et al., 2019), and the high-quality paired-clean reads were then mapped onto the indexed genome. The abundance estimation of gene family members was performed by using StringTie (Pertea et al., 2016). Finally, the heatmap was generated by using the fragments per kilobase of transcript per million mapped reads (FPKM) values.

### 3D structure prediction of TIR1/AFB proteins

A protein requires a three-dimensional (3D) structure to perform its functions properly. The 3D structures of four Dangshansuli TIR1/AFBs (DaAFB1, DaAFB3a, DaAFB3b, and DaAFB5) were predicted by using the AlphaFold2 (https://colab.research.google.com/github/sokrypton/ColabFold/blob/main/AlphaFold2.ipynb) (Jumper et al., 2021). The predicted 3D structures were evaluated by using the SAVES (https://saves.mbi.ucla.edu/) (Zameer et al., 2022) server and MolProbity (http://molprobity.biochem.duke.edu/) (Davis et al., 2007). Finally, the UCSF ChimeraX software (Goddard et al., 2018) was used to visualize these structures (Neupane et al., 2018; Zia et al., 2024).

## Results

### Identification and physiochemical characteristics of TIR1/AFB genes in *Pyrus* genomes

A total of seven genes from the Cuiguan genome (CuTIR1/AFB), six from Shanxi Duli (ShTIR1/AFB), six from Zhongai1 (ZhTIR1/AFB), seven from Nijisseiki (NiTIR1/AFB), six from Yunhong No.1 (YuTIR1/AFB), nine from d’Anjou (AnTIR1/AFB), five from Bartlett v2.0 (BrTIR1/AFB), and four from the Dangshansuli’ v.1.1 genome (DaTIR1/AFB) were identified. All of these members were confirmed for the presence of the F-box_5 superfamily domain (Supplementary Table S3). The protein names of each member were given according to their phylogenetic relationships (Table 1).

**TABLE 1:** TIR1/AFB gene family members identified in eight *Pyrus* genomes, their physicochemical characteristics including protein length (AA), molecular weight (MW), isoelectric point (pI), insatiability index (II), aliphatic index (AI), grand average of hydropathicity (GRAVY), and subcellular localization.

Gene name	Transcript ID	Chr	Start	End	AA	MW (kD)	Pi	II	AI	GRAVY	Subcellular localization
Cuiguan											
CuTIR1	EVM0011146	Chr3	1323365	1326348	584	65.75	6.02	52.66	97.84	−0.026	Nucleus
CuAFB1a	EVM0036646	Chr5	2146830	2150162	568	63.55	5.08	44.72	90.25	−0.091	Chloroplast
CuAFB1b	EVM0021404	Chr11	30922739	30926507	584	65.81	5.90	53.52	96.82	−0.052	Nucleus
CuAFB2	EVM0025311	Chr16	34199	36681	570	63.98	7.41	41.36	99.53	−0.035	Cytoplasm
CuAFB3	EVM0030244	Chr17	899795	902299	572	63.93	5.82	44.08	100.72	−0.013	Chloroplast
CuAFB4	EVM0003039	Chr8	3778806	3781091	614	67.96	5.43	51.35	86.37	−0.097	Nucleus
CuAFB5	EVM0018221	Chr15	5496821	5499124	629	69.90	5.42	50.96	90.21	−0.067	Nucleus
**Shanxi Duli**											
ShTIR1	Chr3.g17722	GWHAAYT00000003	30104995	30108647	584	65.72	5.96	52.30	97.84	−0.023	Nucleus
ShAFB1a	Chr5.g05947	GWHAAYT00000005	35052272	35054657	568	63.58	5.13	46.08	90.25	−0.091	Chloroplast
ShAFB1b	Chr11.g13277	GWHAAYT00000011	33989369	33993093	584	65.84	5.90	53.54	97.14	−0.047	Nucleus
ShAFB2	Chr9.g47408	GWHAAYT00000009	301448	304803	570	63.96	7.41	41.36	99.70	−0.033	Nucleus
ShAFB3	Chr17.g24635	GWHAAYT00000017	322102	325619	572	63.96	5.82	43.66	101.05	−0.009	Chloroplast
ShAFB5	Chr15.g00688	GWHAAYT00000015	39576679	39580197	639	71.08	5.42	51.69	88.79	−0.062	Nucleus
**Zhongai 1**											
ZhTIR1	Pdr3g015830	Chr3	19740066	19743786	584	65.72	6.02	53.53	97.84	−0.021	Nucleus
ZhAFB1a	Pdr11g001830	Chr11	1280166	1283865	584	65.89	6.31	52.98	97.83	−0.052	Cytoplasm
ZhAFB1b	Pdr5g024650	Chr5	28444725	28447965	568	63.45	5.07	44.27	91.44	−0.058	Chloroplast
ZhAFB3	Pdr17g000380	Chr17	315564	319202	572	64.02	6.10	43.71	101.05	−0.020	Nucleus
ZhAFB4a	Pdr8g011790	Chr8	10599351	10602955	632	70.26	5.59	50.13	91.31	−0.007	Nucleus
ZhAFB4b	Pdr8g011760	Chr8	10575346	10578740	632	70.30	5.69	51.41	89.92	−0.050	Nucleus
**Nijisseiki**											
NiTIR1a	Ppy11g2693.1	PPY_r1.0chr11	34498807	34501824	584	65.82	5.90	52.62	96.82	−0.051	Nucleus
NiTIR1b	Ppy03g2593.1	PPY_r1.0chr03	30332146	30335129	584	65.75	6.02	52.66	97.84	−0.026	Nucleus
NiAFB1	Ppy05g3011.1	PPY_r1.0chr05	33363278	33366576	638	71.58	5.15	48.64	91.79	−0.064	Nucleus
NiAFB2	Ppy09g0041.1	PPY_r1.0chr09	286281	288853	600	67.48	8.00	40.59	98.12	−0.052	Chloroplast
NiAFB3	Ppy17g0041.1	PPY_r1.0chr17	308939	311442	572	63.98	5.90	44.78	100.03	−0.035	Nucleus
NiAFB4	Ppy08g2046.1	PPY_r1.0chr08	22153002	22155297	632	70.34	5.61	50.33	90.54	−0.022	Nucleus
NiAFB5	Ppy15g3202.1	PPY_r1.0chr15	34309566	34311870	639	71.01	5.36	51.35	89.56	−0.050	Nucleus
**Yunhong No.1**											
YuTIR1a	Pspp.Chr11.02380	Chr11	31780785	31784509	584	65.81	5.90	53.52	96.82	−0.052	Nucleus
YuTIR1b	Pspp.Chr03.02292	Chr3	28530646	28534264	584	65.75	6.02	52.66	97.84	−0.026	Nucleus
YuAFB2	Pspp.Chr09.00040	Chr9	284261	287834	570	63.98	7.41	41.36	99.53	−0.035	Nucleus
YuAFB3	Pspp.Chr17.00041	Chr17	289969	293508	572	63.98	5.90	44.78	100.03	−0.035	Chloroplast
YuAFB4	Pspp.Chr08.01596	Chr8	20332504	20335583	632	70.42	5.61	50.02	90.54	−0.022	Nucleus
YuAFB5	Pspp.Chr15.03296	Chr15	36126536	36130377	639	71.02	5.42	51.21	89.56	−0.051	Nucleus
**d’Anjou**											
AnTIR1a	DAnjou_Chr3v0.1_07867	Chr3	30202897	30206335	584	65.75	6.02	52.66	97.84	−0.026	Nucleus
AnTIR1b	DAnjou_Chr11v0.1_29125	Chr11	37463591	37467089	584	65.82	6.15	53.04	96.82	−0.060	Nucleus
AnAFB1	DAnjou_Chr5v0.1_13247	Chr5	37008128	37010513	568	63.62	5.17	44.99	90.76	−0.083	Chloroplast
AnAFB2a	DAnjou_Chr17v0.1_42902	Chr17	1482299	1485079	622	69.98	7.42	40.55	98.89	−0.045	Cytoplasm
AnAFB2b	DAnjou_Chr17v0.1_42928	Chr17	1636041	1638819	622	69.98	7.42	39.29	98.89	−0.045	Chloroplast
AnAFB3	DAnjou_Chr17v0.1_42725	Chr17	281859	284630	612	68.63	6.34	42.98	98.12	−0.044	Nucleus
AnAFB4a	DAnjou_Chr8v0.1_20617	Chr8	21228650	21231853	632	70.26	5.59	50.13	91.31	−0.007	Nucleus
AnAFB4b	DAnjou_Chr11v0.1_27369	Chr11	10024141	10027113	632	70.29	5.58	50.20	91.77	−0.009	Nucleus
AnAFB5	DAnjou_Chr15v0.1_39634	Chr15	39513526	39516783	639	70.97	5.29	51.33	90.17	−0.044	Nucleus
**Bartlett**											
BrTIR1	pycom11g26260	Chr11	28868074	28870429	367	41.39	7.45	54.99	96.98	−0.026	Nucleus
BrAFB1	pycom05g30000	Chr5	30268376	30270762	568	63.45	5.07	44.27	91.44	−0.058	Chloroplast
BrAFB2	pycom111g00370	SuperScaffold	271984	274466	570	64.03	7.41	39.57	99.70	−0.039	Nucleus
BrAFB4	pycom08g16580	Chr8	16549295	16551589	632	70.31	5.52	50.02	90.85	−0.016	Cytoplasm
BrAFB5	pycom15g34120	Chr15	33696788	33699094	639	70.97	5.29	51.33	90.17	−0.044	Nucleus
**Dangshansuli**											
DaAFB1	LOC103963931	Chr5	25022267	25025544	568	63.51	5.08	44.57	89.74	−0.099	Nucleus
DaAFB3a	LOC103966576	Chr17	21746650	21749990	572	64.08	6.04	43.46	100.37	−0.018	Chloroplast
DaAFB3b	LOC103936090	Chr17	19463819	19467247	572	63.98	5.90	44.78	100.03	−0.035	Chloroplast
DaAFB5	LOC103962651	Chr15	35970186	35973742	639	71.06	5.42	50.91	90.33	−0.042	Nucleus

The length of the Cuiguan TIR1/AFB proteins ranged from 584aa–629aa and MW: 63 kDa–69 kDa. The CuTIR1/AFBs were located in the nucleus, chloroplast, and cytoplasm. For Shanxi Duli, the length of ShTIR1/AFB proteins ranged from 568aa–639aa and MW: 62 kDaa–71 kDa. The ShTIR1/AFBs were located in the nucleus and chloroplast. For Zhongai 1, the length of ZhTIR1/AFB proteins ranged from 568aa–632aa, MW: 63 kDa–70 kDa. The ZhTIR1/AFBs were located in the nucleus, chloroplast, and cytoplasm. For Nijisseiki, the length of NiTIR1/AFB proteins ranged from 572aa–639aa, MW: 63 kDa–71 kDa. The NiTIR1/AFBs were located in the nucleus and chloroplast. For Yunhong No. 1, the length of YuTIR1/AFB proteins ranged from 570aa–639aa and MW: 63 kDa–71 kDa. The YuTIR1/AFBs were located in the nucleus and chloroplast. For d’Anjou, the length of AnTIR1/AFB proteins ranged from 568aa–639aa and MW: 65 kDa–70 kDa. The AnTIR1/AFBs were located in the nucleus, cytoplasm, and chloroplast. For Bartlett, the length of BrTIR1/AFB proteins ranged from 568aa–639aa and MW: 41 kDa–70 kDa. The BrTIR1/AFBs were located in the nucleus, cytoplasm, and chloroplast. For Dangshansuli, the length of DaTIR1/AFB proteins ranged from 568aa–639aa and MW: 63 kDa–71 kDa. The DaTIR1/AFBs were located in the nucleus and chloroplast.

These results show that the members did not show significant differences in aa and MW values except for some proteins that showed deviant behavior. The *Pyrus* TIR1/AFB proteins had pI values ranging from 5–8, indicating their acidic as well as basic behavior. The II values of all these proteins were above 40, which indicates that these are unstable in the test tube. The AI values of all the *Pyrus* TIR1/AFB proteins indicated that these proteins are thermally stable. Furthermore, the negative GRAVY values of all these proteins indicated that these proteins are hydrophilic (Table 1).

### Phylogenetic relationships of *Pyrus* TIR1/AFB family members

**FIGURE 1:**
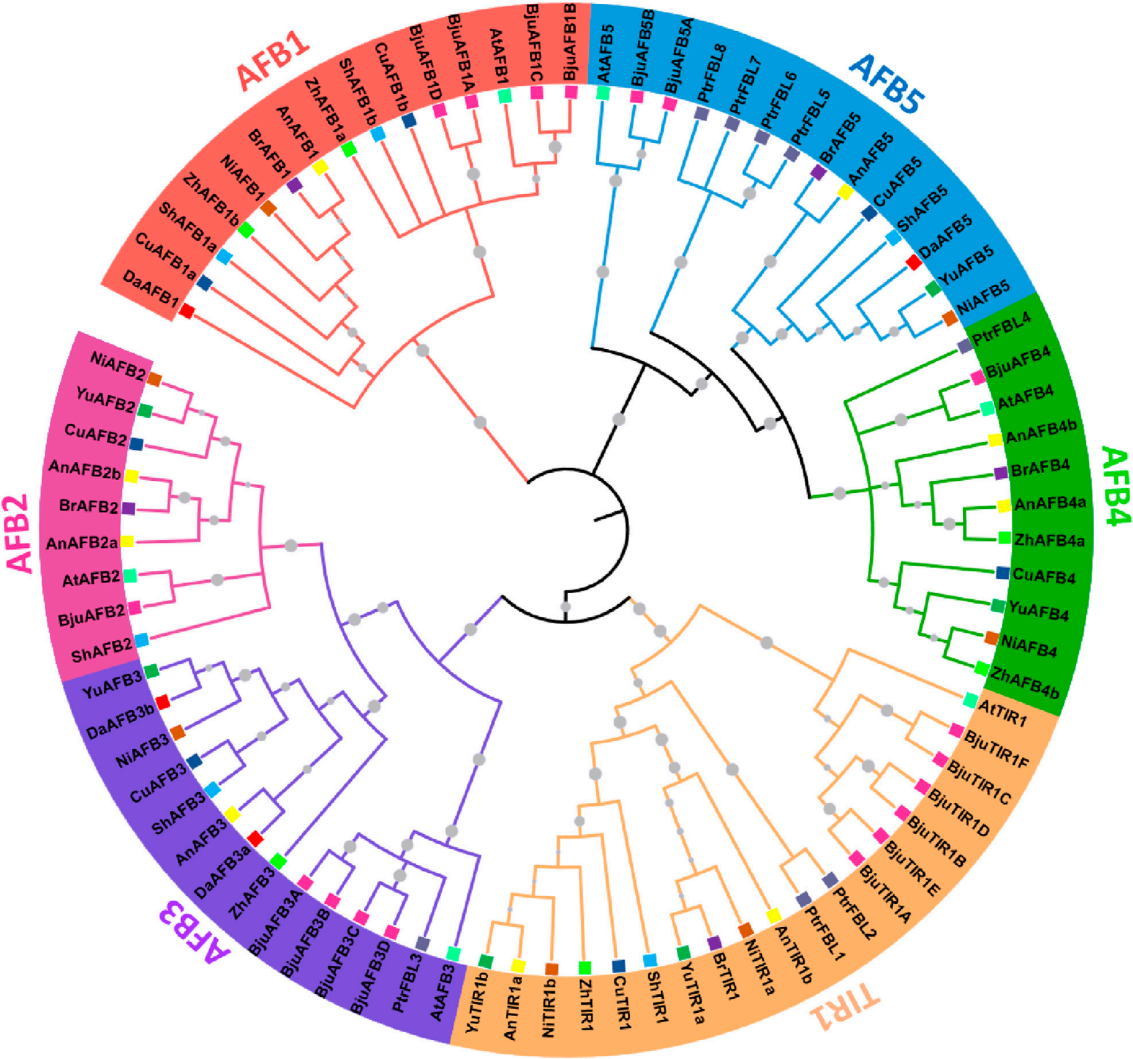
A maximum likelihood (ML) phylogenetic tree with 1000 bootstrap replicates (denoted by gray circle symbols) was generated using TIR1/AFB members from eight *Pyrus*, *A. thaliana*, *B. juncea*, and *Populus* genomes. Different branch colors represent different groups. Furthermore, each species is given a particular-colored square symbol at the leaf end.

To analyze the evolutionary relationships among the TIR1/AFB members from eight *Pyrus* genomes, a phylogenetic tree was constructed by using 78 amino acid sequences from 11 species: eight *Pyrus*, *A. thaliana*, *B. juncea*, and Populus. All TIR1/AFB proteins were clustered into six groups: TIR1, AFB1, AFB2, AFB3, AFB4, and AFB5. TIR1 comprised the largest clade containing one AtTIR1, and the remaining members belonged to *Pyrus* genomes. AFB1 contained members from all eight *Pyrus* genomes. The AFB2 clade contained members from seven genomes but no members from the Zhongai1 genome. The AFB3 clade contained members from all seven genomes and no members from the Bartlett genome. The AFB4 clade contained members from six genomes, except the Shanxi Duli and Dangshansuli genomes. The AFB5 clade contained members from seven *Pyrus* genomes with no members from the Zhongai1 genome (Figure 1).

Each group is represented by a particular color, and specific colors are used for each species’ genome.

### Conserved motifs and gene structure analysis of *Pyrus* TIR1/AFBs

**FIGURE 2:**
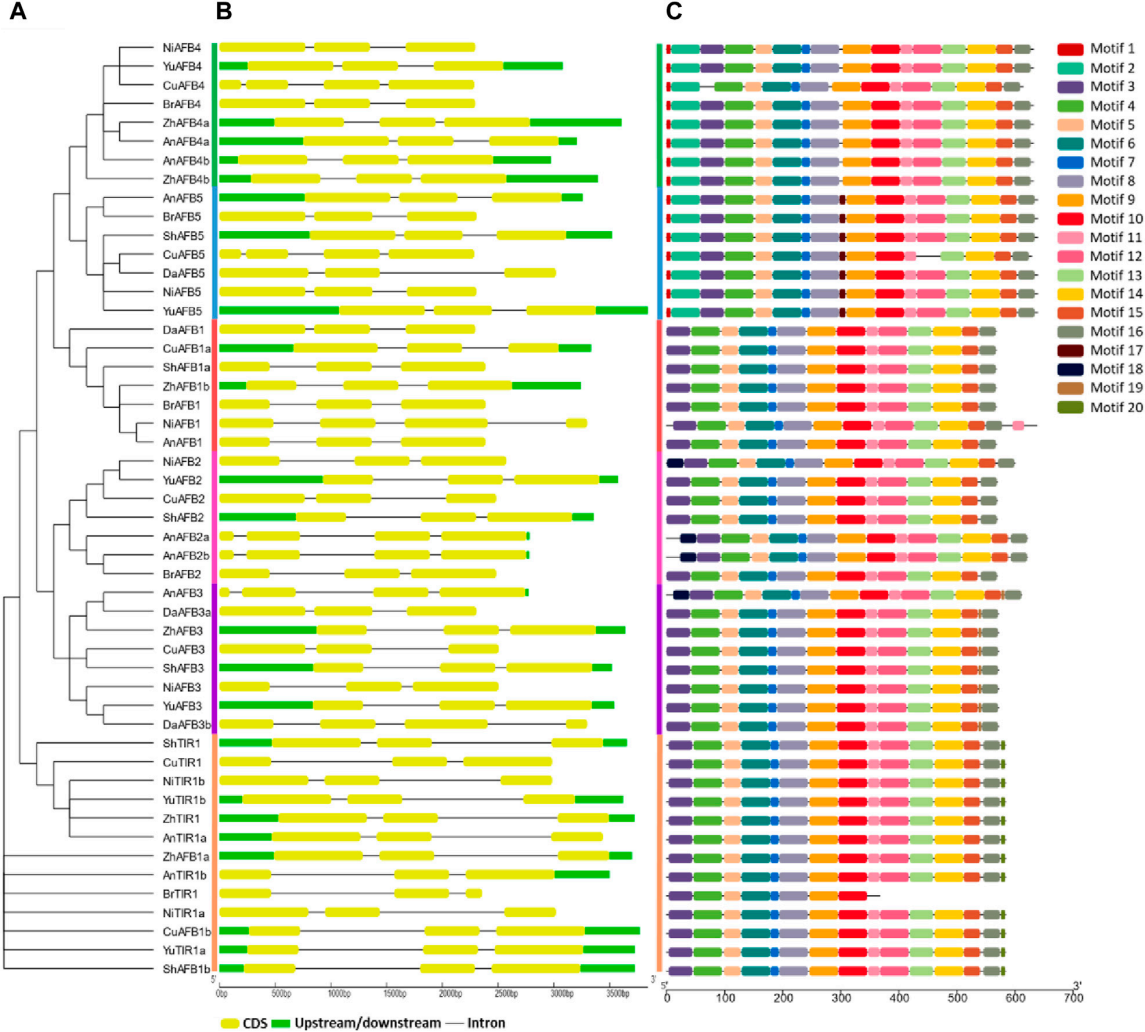
**(A)** Phylogenetic tree, motif pattern, and gene structures of Pyrus TIR1/AFBs, **(B)** gene structure showing conservation pattern of exons and introns, and **(C)** conserved motifs determined using the MEME suits.

The conserved motifs and gene structures were analyzed to reveal the evolutionary pattern among *Pyrus* TIR1/AFBs. All members of each subfamily shared highly conserved motifs. Members of TIR1, AFB1, AFB2, and AFB3 have exactly similar motif patterns (conserved motifs 3–16). Meanwhile, only one member of the Bartlett genome, BrTIR1, had a different conservation pattern, with only motifs 3–10 being conserved. Members belonging to groups AFB4 and AFB5 showed a similar pattern as the groups mentioned earlier with some other motifs (motifs 1 and 2) (Figure 2A,C). This high conservation of motifs implies no major differences in the structure and functions of *Pyrus* TIR1/AFBs.

Similarly, the gene structure was also found to be highly conserved among the members of the same subfamily. Almost all members contained at least three exons and two introns. A few members of the AFB2 subfamily (AnAFB2a, AnAFB2b, and AnAFB3) contained four conserved exons with three introns (Figure 2B). This conservation of motifs and exons among the *Pyrus* TIR1/AFBs indicated that these genes are conserved across eight *Pyrus* genomes.

### Chromosomal mapping and gene duplication analysis

**FIGURE 3:**
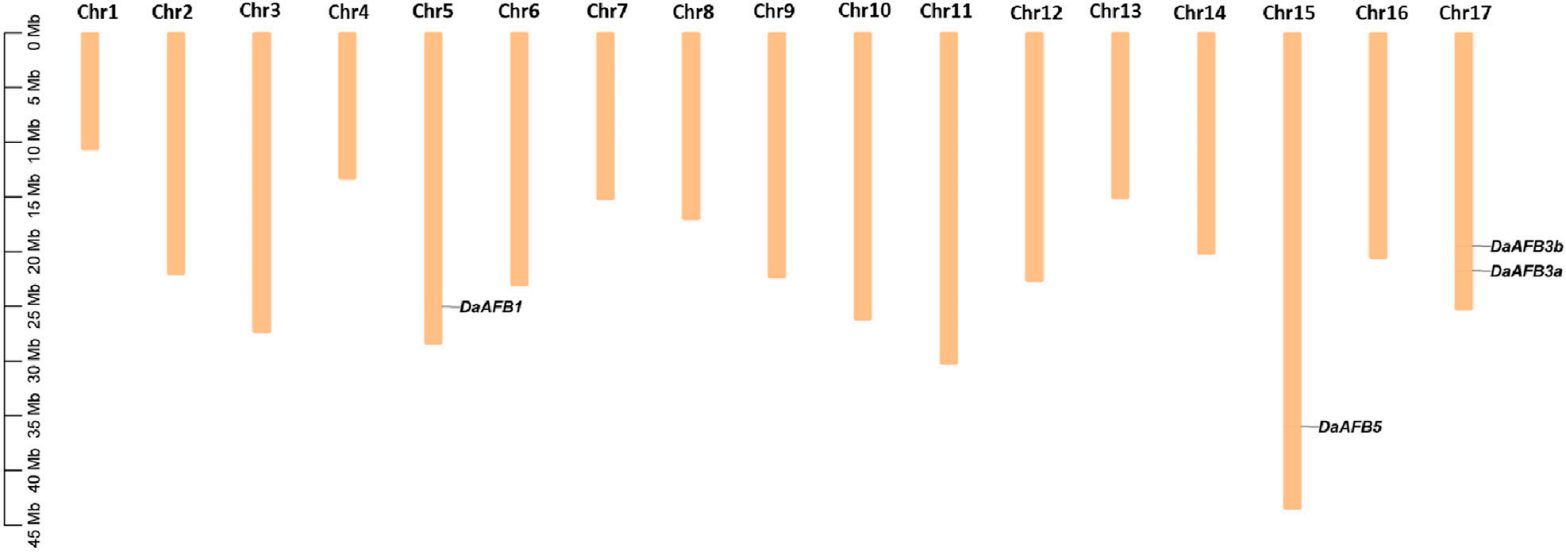
Chromosomal map showing four *DaTIR1/AFBs* distributed on 3 of 17 Dangshansuli’ v.1.1 chromosomes.

The chromosomal gene localization was identified to evaluate the gene distribution pattern of *Pyrus TIR1/AFBs* on 17 chromosomes of each *Pyrus* genome. This analysis showed that all genes were unevenly distributed on chromosomes. In the Cuiguan genome, seven *CuTIR1/AFBs* were found to be localized on seven of seventeen chromosomes (Chr3, 5, 8, 11, 15, 16, and 17). The remaining chromosomes had no *CuTIR1/AFB* genes present on them. In Shanxi Duli, six *ShTIR1/AFBs* were scattered on six of seventeen chromosomes. In the Zhongai1 genome, six *ZhTIR1/AFBs* were distributed on five of seventeen chromosomes. In the Nijisseiki genome, seven *NiTIR1/AFBs* were distributed on seven chromosomes. In the Yunhong No.1 genome, six *YuTIR1/AFBs* were unevenly distributed on six chromosomes. Seven *AnTIR1/AFB*s in d’Anjou genomes were distributed on six chromosomes. Five Bartlett v2.0 *BrTIR1/AFBs* were distributed on four chromosomes (Supplementary Figures S1–S7). Four *DaTIR1/AFB* members from the Dangshansuli’ v.1.1 genome were distributed on 3 of 17 chromosomes (Figure 3).

Gene duplication events were also analyzed among members of each Pyrus *TIR1/AFBs* (Table 2). The Cuiguan genome contained four duplicated pairs of genes, with all of them being segmentally duplicated. The genome of Shanxi Duli contained five segmentally duplicated pairs of Sh*TIR1/AFBs*. The Zhongai1 genome contained four duplicated pairs of Zh*TIR1/AFBs*. Of these four pairs, one (*ZhAFB4a/ZhAFB4b*) exhibited tandem duplication, while the rest showed segmental duplication. The Nijisseiki genome contained five pairs of segmentally duplicated Ni*TIR1/AFBs*. The Yunhong No.1 genome contained four pairs of *YuTIR1/AFB* genes that originated through segmental duplication. The d’Anjou genome exhibited eight pairs of duplicated genes. Of these two pairs, *AnAFB2a*/*AnAFB3* and *AnAFB2b*/*AnAFB3* had tandem duplication, while the rest were segmentally duplicated. The Bartlett genome contained two pairs of segmentally duplicated genes, *BrTIR1*/*BrAFB2* and *BrAFB4*/*BrAFB5*. The Dungshanxuli genome exhibited only one segmentally duplicated gene pair, *DaAFB1*/*DaAFB3a*.

**TABLE 2:** Duplication data on *Pyrus TIR1/AFBs*, rate of synonymous (Ka) and non-synonymous mutations (Ks), duplication time (MYA), and the type of duplication.

*Pyrus* genome	Gene 1	Gene 2	Ka/Ks	Duplication time (MYA)	Duplication type
Cuiguan	*CuTIR1*	*CuAFB1b*	1.04	3.25	Segmental
	*CuTIR1*	*CuAFB3*	0.46	90.83	Segmental
	*CuAFB1b*	*CuAFB3*	0.54	76.89	Segmental
	*CuAFB2*	*CuAFB3*	1.14	2.92	Segmental
Shanxi Duli	*ShTIR1*	*ShAFB1b*	0.66	61.52	Segmental
	*ShTIR1*	*ShAFB3*	0.60	81.74	Segmental
	*ShAFB1b*	*ShAFB2*	0.88	28.83	Segmental
	*ShAFB1b*	*ShAFB3*	0.96	26.41	Segmental
	*ShAFB2*	*ShAFB3*	0.86	3.74	Segmental
Zhongai1	*ZhTIR1*	*ZhAFB1a*	0.77	3.80	Segmental
	*ZhTIR1*	*ZhAFB3*	0.91	72.09	Segmental
	*ZhAFB1a*	*ZhAFB3*	0.79	82.13	Segmental
	*ZhAFB4a*	*ZhAFB4b*	0.74	0.78	Tandem
Nijisseiki	*NiTIR1a*	*NiTIR1b*	1.07	2.76	Segmental
	*NiTIR1a*	*NiAFB3*	0.30	138.16	Segmental
	*NiTIR1b*	*NiAFB3*	0.46	94.98	Segmental
	*NiAFB2*	*NiAFB3*	0.87	3.54	Segmental
	*NiAFB4*	*NiAFB5*	0.89	2.71	Segmental
Yunhong No.1	*YuTIR1a*	*YuAFB3*	0.67	34.93	Segmental
	*YuTIR1b*	*YuAFB3*	0.59	94.56	Segmental
	*YuAFB2*	*YuAFB3*	1.01	3.27	Segmental
	*YuAFB4*	*YuAFB5*	0.86	3.40	Segmental
d’Anjou	*AnTIR1b*	*AnAFB2a*	0.80	27.84	Segmental
	*AnTIR1b*	*AnAFB2b*	0.80	27.92	Segmental
	*AnTIR1b*	*AnAFB3*	0.84	26.39	Segmental
	*AnAFB2a*	*AnAFB3*	0.76	3.78	Tandem
	*AnAFB2b*	*AnAFB3*	0.77	3.79	Tandem
	*AnAFB4a*	*AnAFB4b*	0.98	57.99	Segmental
	*AnAFB4a*	*AnAFB5*	0.89	2.39	Segmental
	*AnAFB4b*	*AnAFB5*	0.93	61.78	Segmental
Bartlett	*BrTIR1*	*BrAFB2*	0.84	32.07	Segmental
	*BrAFB4*	*BrAFB5*	1.01	2.25	Segmental
Dungshanxuli	*DaAFB1*	*DaAFB3a*	0.58	3.60	Segmental

To analyze the evolutionary constraints of the repeated Pyrus TIR1/AFB genes, the Ka, Ks, and Ka/Ks ratios of all para-homologous gene pairs were also calculated. The Ka/Ks ratio ranged from 0.5 to 1.1 in the eight genomes, which shows both positive and negative selection events. The time of divergence of all 30 duplicated gene pairs of Pyrus TIR1/AFBs was between 0.7 and 90.83 million years ago (MYA), which suggests a significant period of evolutionary divergence (Table 2).

### PPI and GO enrichment analyses

**FIGURE 4:**
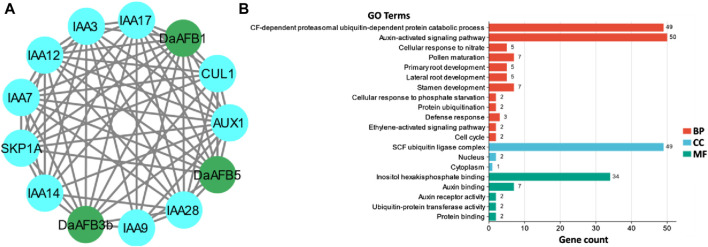
**(A)** Interactions among Pyrus TIR1/AFBs (shown in dark green nodes) and other homologous proteins (shown in blue color nodes). Gray lines indicate the interactions. **(B)** Predicted biological processes (BP), cellular components (CC), and molecular functions (MF) associated with *Pyrus TIR1/AFBs*.

A PPI network of the Pyrus TIR1/AFBs was generated to understand the functional diversity among members. The three Dangshansuli members, DaAFB1, DaAFB3b, and DaAFB5, interacted with several other proteins. Most of the interactions were identified with 1AA and AUX proteins, which shows the potential roles of these Pyrus members in auxin regulation, thus their involvement in the growth and development of plants (Figure 4A).

GO enrichment analysis was carried out to ascertain the molecular roles of Pyrus TIR1/AFBs in a dynamic manner. Based on this GO analysis, Pyrus TIR1/AFB genes were classified into three different major categories: BPs, CCs, and MFs. Major BPs predicted included auxin-activated signaling pathways, defense responses, and other developmental and signaling pathways. These genes were found in CCs, including the nucleus, cytoplasm, and SCF ubiquitin ligase complex. The MFs associated with these genes included auxin and protein binding activity (Figure 4B; Supplementary Table S2). All these terms clearly indicate the functional involvement of Pyrus *TIR1/AFBs* in auxin signaling and drought stress responsiveness, thus resulting in growth.

### *Cis*-regulatory element analysis of *Pyrus TIR1/AFBs*

**FIGURE 5:**
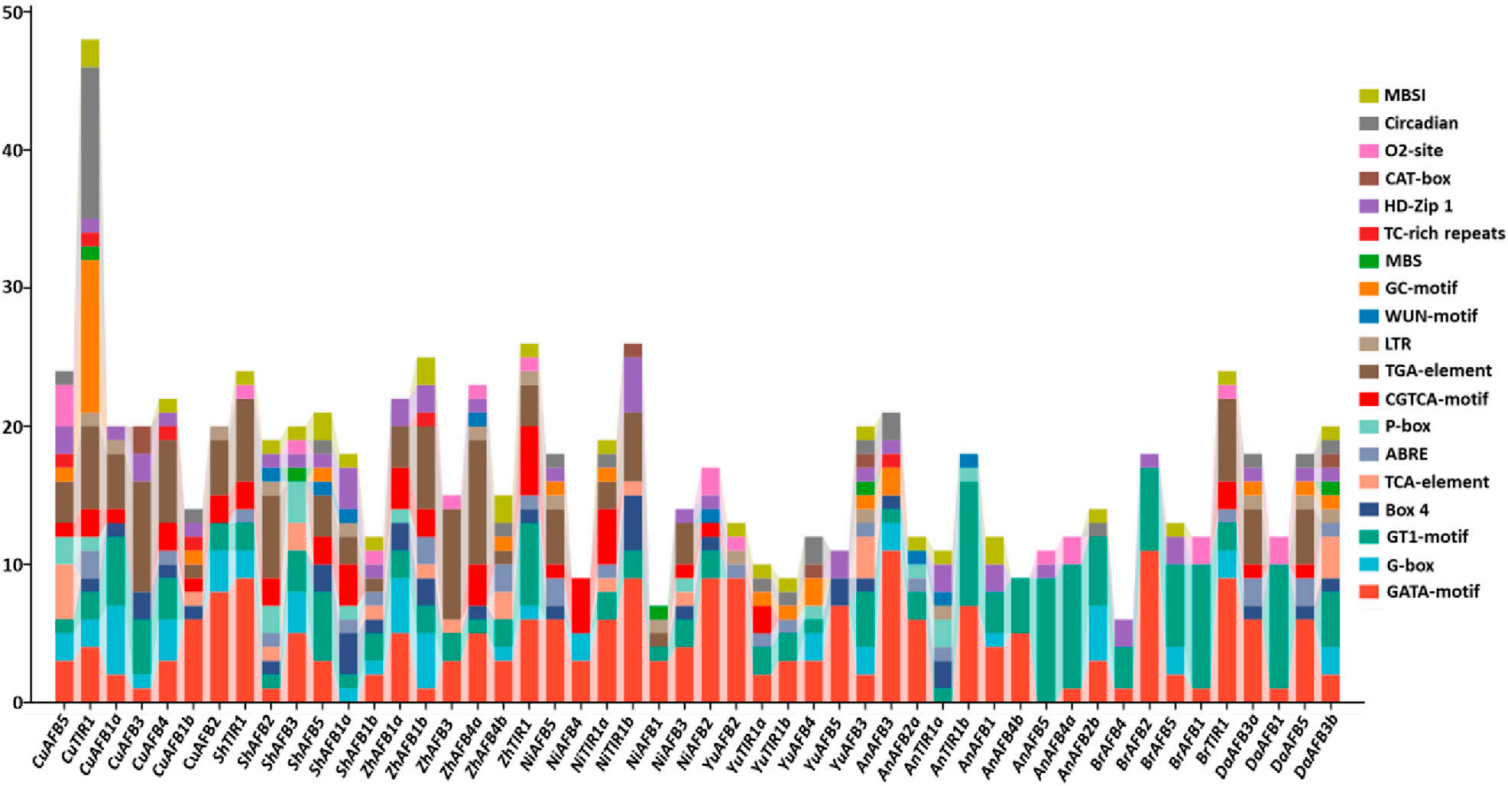
The Pyrus *TIR1/AFB* genes’ upstream promoter regions contain *cis*-regulatory elements. Every bar represents a distinct element found in a given gene.

To gain better insights into the diverse stress responses of Pyrus *TIR1/AFBs*, *cis*-regulatory elements in their promoter sequences were analyzed. In all genomes, *cis* elements associated with stress, such as light, hormones, and development-related responsiveness, were abundantly found. In relation to these elements, it was discovered that G-box, GT1-motif, and GATA-motif—*cis* elements Box 4—were implicated in the regulation of light stress. Hormone responsiveness was linked to five *cis* elements: P-box, TGA-element, ABRE, CGTCA-motif, and TCA-element. Furthermore, it was discovered that the GC-motif, LTR, TC-rich repeats, and MBS are the four *cis* elements associated with stress responsiveness. Developmental processes involved five elements: CAT-box, MBSI, circadian, HD-Zip 1, and o2-site. In *Pyrus TIR1/AFBs*, the presence of these elements shows their hormones, stress, and development-related responses (Figure 5; Supplementary Table S1).

### Expression profiling of *Pyrus TIR1/AFBs*

**FIGURE 6:**
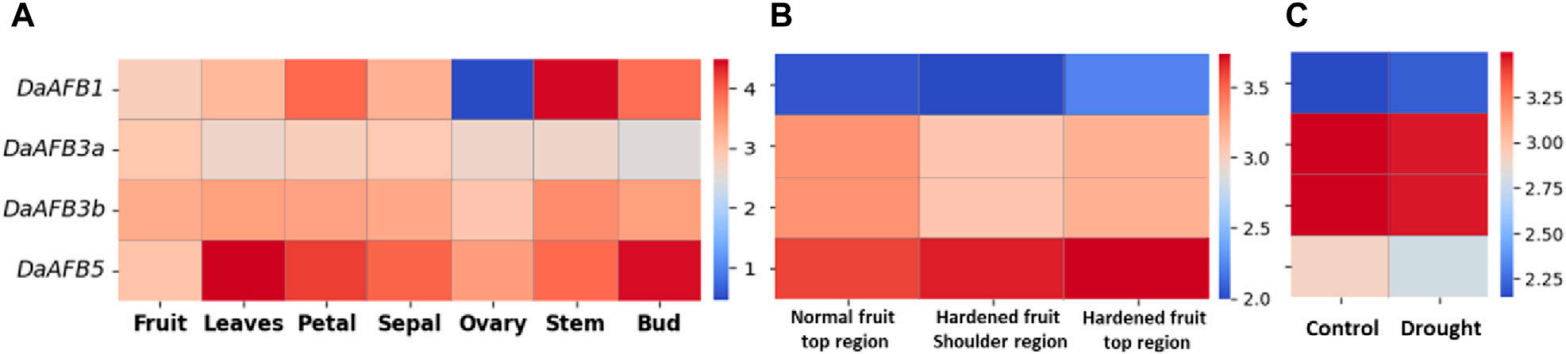
Heatmap showing the expression pattern of *DaTIR1/AFBs* in **(A)** different tissues where blue shows a decreased expression downregulation and red shows an increased expression, **(B)** disease condition where the normal fruit region is compared with hardened fruit, and **(C)** drought stress condition.

Transcriptome expression data were used to determine the expression level of four *DaTIR1/AFBs* in tissues, including fruit, leaves, petals, sepals, ovaries, stems, and buds. *DaAFB1* showed a decline in expression in ovary tissues. However, it was highly expressed in stem tissues. *DaAFB5* exhibited a higher expression in leaves and bud tissues (Figure 6A).

Expression profiles of *DaTIR1/AFBs* in fruit hardening disease conditions were also performed. *DaAFB1* was highly downregulated under disease conditions. *DaAFB5* showed an increased expression. However, *DaAFB3a/3b* showed no significant change in expression (Figure 6B). Under drought stress, *DaAFB1* showed a large decrease in expression. *DaAFB3a/3b* showed overexpression in drought stress, and *DaAFB5* exhibited no specific change in expression (Figure 6C). These results show the differential expression pattern at various stages and biotic and abiotic stress conditions.

### 3D structure prediction of DaTIR1/AFB proteins

**FIGURE 7:**
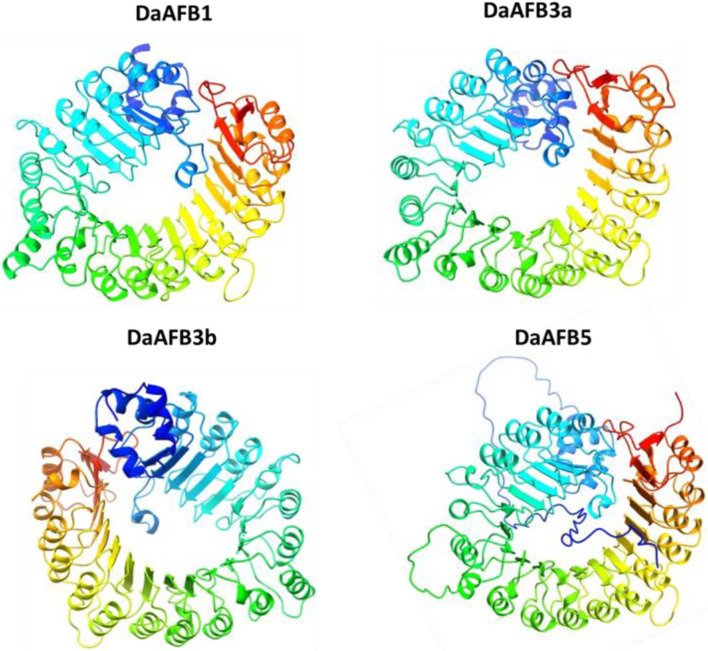
Predicted 3D structures of four DaTIR1/AFB proteins. In structures, blue and cyan colors represent the helices; yellow-green color patterns represent the turns.

To gain further insights into the structural and functional diversity, the 3D structures of the four DaTIR1/AFB proteins were modeled. The four proteins showed high conservation in 3D structures. All these four proteins contained similar structures of helices and turns. All the proteins shared a similar helical structure at the C and N termini. This conservation of structure suggests the potential similar functions of DaTIR1/AFB proteins as the *DaAFB1* showed similar expression results in ovary tissues, fruit hardening, and drought stress conditions. Similarly, *DaAFB5* also showed conservation in expression levels in various tissues and disease conditions (Figure 7).

## Discussion

TIR1/AFB proteins have critical roles in auxin signaling. In *Arabidopsis*, these gene family members comprise a group of related proteins that include TIR1 and AFB1/2/3/4/5. Previous studies have reported that TIR1, along with AFBs, functions to regulate auxin signaling, which ultimately impacts plant growth and development (Dharmasiri et al., 2005). The present study reports a pangenome-wide identification of the TIR1/AFB gene family in eight *Pyrus* genomes. The TIR1/AFB gene family has already been reported in Arabidopsis, where the TIR1/AFB gene showed a significant role in lateral root development and hypocotyl elongation (Ruegger et al., 1998). In *B. juncea* var. *tumida* (Cai et al., 2000), 18 members showed differential expression in different tissues and salt stress. Similarly, eight members of this gene family have been reported in the *Populus* genome, where *TIR1s* showed differential expression in stem growth and drought stress (Shu et al., 2015). Moreover, this gene family has also been analyzed in *Physcomitrium patens*, *Fragaria vesca* (Fatima et al., 2023b)*,* and *Selaginella moellendorffii* genomes (Su et al., 2023b), where these genes showed the tissue-specific expression patterns. These results suggest that this gene family contributes to plant development and responses to abiotic stresses.

The phylogenetic tree revealed that *Pyrus* TIR1/AFB proteins are divided into six subfamilies, namely, TRI1, AFB1, AFB2, AFB3, AFB4, and AFB5. This division of clades is consistent with other species, including *Arabidopsis* and Populus. In *B. juncea*, the BjuTIR1/AFBs are also divided into the same six clusters of subfamilies. The specific functions of these gene family members vary across and within the clades. For instance, the AFB4 and AFB5 shared the same clade but showed distinct specificities for auxin (Prigge et al., 2020). The tree showed that AFB1s and TIR1s clustered on the individual clades, while AFB2 and AFB3 originated from the same clade and diverged into two subclades, thus expanding independently. Similarly, AFB4 and AFB5 also arose from the same clade and then evolved separately (Dharmasiri et al., 2005). Most of the Pyrus TIR1/AFB proteins contained three conserved exons with two introns except for *BjuAFB1D* and *BjuAFB1D,* which contained four exons with three introns. This pattern of exon-intron conservation aligns with the *Pyrus* members that also contain three exons. Similarly, members of this gene family also showed comparable results in rice (Wang et al., 2007) and maize (Xing et al., 2011) genomes. However, members belonging to AFB4 and AFB5 clades had motifs 2 and 3 conserved. Similarly, *Arabidopsis* AFB2, AFB3, AFB4, and AFB5 showed certain conserved motifs (Du et al., 2022). This presence and absence of certain motifs indicates that TIR1/AFBs may have different functions.

The evolution of the members of this gene family was examined by comparing Pyrus TIR1/AFBs. It was found that almost all genes evolved through segmental duplication. Only one member from the Zhongai1 genome and two from the d’Anjou genome showed tandem duplication. The four Populus gene pairs (*PtrFBL1/PtrFBL2*, *PtrFBL3/PtrFBL4*, *PtrFBL5/PtrFBL6*, and *PtrFBL7/PtrFBL8*) were found to be originated from the genome duplication. These members also exhibited a similar pattern of intensive segmental recombination (Shu et al., 2015). Similarly, the multiple auxin receptor homologs in *B. juncea* also originated due to genome duplication (Cai et al., 2000). However, TIR1/AFBs from land plants exhibited tandem duplication to be more dominant during genome evolution (Su et al., 2023b).

In the promoter regions, *cis* elements were predicted to gain a better understanding of the role of Pyrus *TIR1/AFBs* under various environmental conditions. Similar to land plants P. patens, *S. moellendorfii*, *A. thaliana*, and *F. vesca*, these gene family members contained a large number of hormone-responsive elements (auxin, salicylic acid, gibberellin, and abscisic acid). Moreover, elements related to growth and drought stress were also found (Su et al., 2023a). The promoter of *BjuTIR1/AFB* genes also contained elements related to plant responses to biotic as well as abiotic stress (Cai et al., 2000). Furthermore, the functional prediction through PPI and GO analysis also showed the conservation and involvement of these genes in auxin responsiveness and stress tolerance mechanisms. PPI analysis revealed that *DaAFB1, DaAFB5,* and *DaAFB3b* showed high interactions with IAA proteins. Studies have shown that the IAA proteins help in mediating tolerance to stresses (such as drought) in plants (Salehin et al., 2019). These *DaAFBs* have been shown to be involved in BPs such as auxin-activated signaling pathways and lateral root development processes. The *Arabidopsis AFB1* has been shown to have a specialized function in rapid auxin-dependent inhibition of root growth and the early phase of root gravitropism (Prigge et al., 2020). Thus, the Pyrus *TIR1/AFBs* can be deemed to be potentially involved in abiotic and biotic stress responses mediated by auxin.

RNA-seq expression data analysis revealed that Pyrus *TIR1/AFB* genes express differently in different tissues, fruit hardening disease, and drought stress conditions. Drought stress affects the expression of multiple TIR1/AFB genes, indicating a potential role for the TIR1/AFB family in the drought tolerance pathway (Du et al., 2022). In *Arabidopsis*, the *TIR1* showed an increased expression under drought stress (Benny et al., 2019). The rice *TIR1* and *AFB2* showed decreased expression under drought stress (Sharma et al., 2018). In wheat, the roots exposed to drought stress showed an increase in expression of *AFB2*, suggesting its key role in drought stress responsiveness (Dalal et al., 2018). Due to the targeting and reduction of *AsAFB2* and *AsTIR1* expression, creeping bentgrass (*Agrostis stolonifera* L.) overexpressing the rice pri-miR393a showed increased tolerance to drought stress. DaAFB5 was highly expressed in tissues, while DaAFB1 exhibited low expression. The *BjuTIR1/AFBs* also showed differential expression patterns in different tissues, including root, stem, leaf, flower, pod, and swollen stem. In contrast, these gene family members had halted expression in salt stress (Cai et al., 2000). Promoter, PPI, GO, and expression analyses revealed that *DaTIR1/AFBs* are involved in auxin-mediated stress responsiveness to both abiotic and biotic stresses. Therefore, these genes can be used in future breeding studies to improve Pyrus stress resilience to various environmental stresses.

## Conclusion

The present study provides a systematic as well as comparative analysis of TIR1/AFB genes in eight economically important and nutritious Pyrus genomes. A total of seven genes from the Cuiguan genome (CuTIR1/AFB), six from Shanxi Duli (ShTIR1/AFB), six from Zhongai1 (ZhTIR1/AFB), seven from Nijisseiki (NiTIR1/AFB), six from Yunhong No.1 (YuTIR1/AFB), nine from d’Anjou (AnTIR1/AFB), five from Bartlett v2.0 (BrTIR1/AFB), and four from the Dangshansuli’ v.1.1 genome (DaTIR1/AFB) were identified. These genes were classified into six groups. All the members from the same group shared conservation in motif and gene structure. Most of the genes originated through segmental duplication, and a few originated through tandem duplication. A large number of *cis* elements related to hormones, stress, and development were found in the promoter region of these *Pyrus TIR1/AFBs*. PPI and GO also revealed the involvement of these genes in auxin-mediated stress-related and development-related processes. Structure prediction showed high conservation among members of *DaTIR1/AFBs*. *DaTIR1*, *DaTIR3a*, *DaTIR3b*, and *DaTIR5* showed differential expression in various tissues, fruit hardening disease, and drought stress. Our results provide a solid foundation to further investigate the function of TIR1/AFBs in regulating various abiotic and environmental stress responses in *Pyrus*.

## Data Availability

The original contributions presented in the study are included in the article/Supplementary Material; further inquiries can be directed to the corresponding authors.

## Supplementary material

SUPPLEMENTARY FIGURE S1
*CuTIR1/AFBs* distributed on Cuiguan chromosomes.

SUPPLEMENTARY FIGURE S2
*ShTIR1/AFBs* distributed on Shanxi Duli chromosomes.

SUPPLEMENTARY FIGURE S3
*ZhTIR1/AFBs* distributed on Zhongai1 chromosomes.

SUPPLEMENTARY FIGURE S4
*NiTIR1/AFBs* distributed on Nijisseiki chromosomes.

SUPPLEMENTARY FIGURE S5
*YuTIR1/AFBs* distributed on Yunhong No.1 chromosomes.

SUPPLEMENTARY FIGURE S6
*AnTIR1/AFBs* distributed on d’Anjou chromosomes.

SUPPLEMENTARY FIGURE S7
*BrTIR1/AFBs* distributed on Bartlett chromosomes.

SUPPLEMENTARY TABLE S1 Identified *cis* elements in *Pyrus TIR1/AFB* genes.

SUPPLEMENTARY TABLE S2 Gene ids, their GO enrichment classes, and description of predicted GO terms.

SUPPLEMENTARY TABLE S3 Domains identified in *Pyrus* TIR1/AFB members.
