# Supplementary material for: Comparative genomic profiling of transport inhibitor Response1/Auxin signaling F-box (TIR1/AFB) genes in eight Pyrus genomes revealed the intraspecies diversity and stress responsiveness patterns
Source: Front Genet. 2024 May 10;15:1393487. doi: 10.3389/fgene.2024.1393487 (PMC11116618; doi:10.3389/fgene.2024.1393487)
Supplement: Supplementary file 1 [file Table1.DOCX]

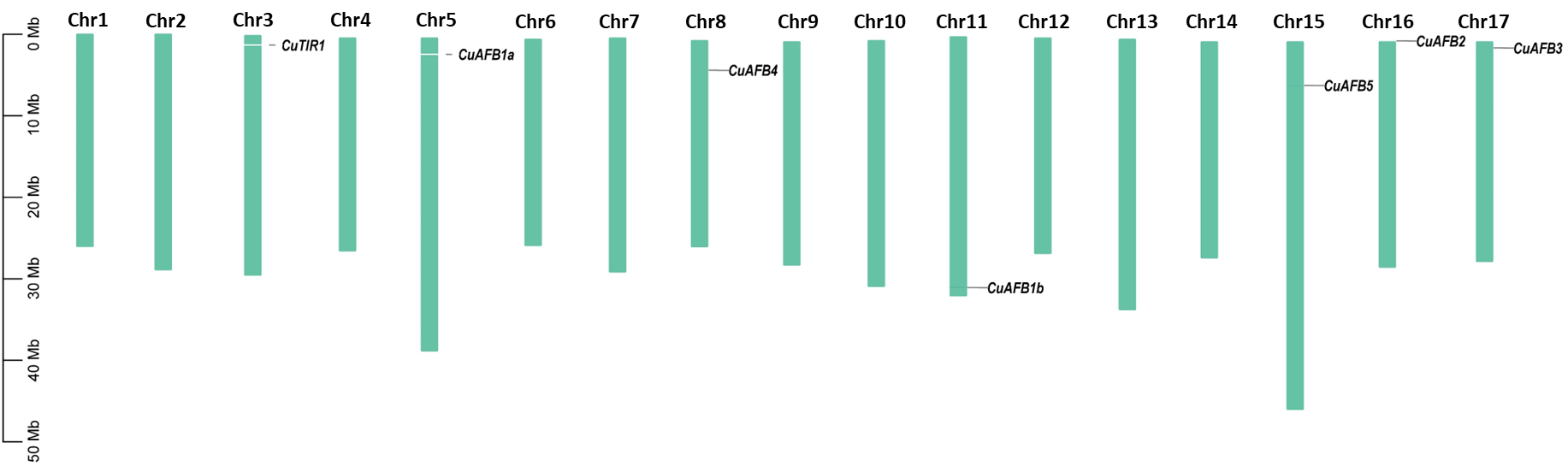


**Supplementary Figure 1:** *CuTIR1/AFBs* distributed on Cuiguan chromosomes.


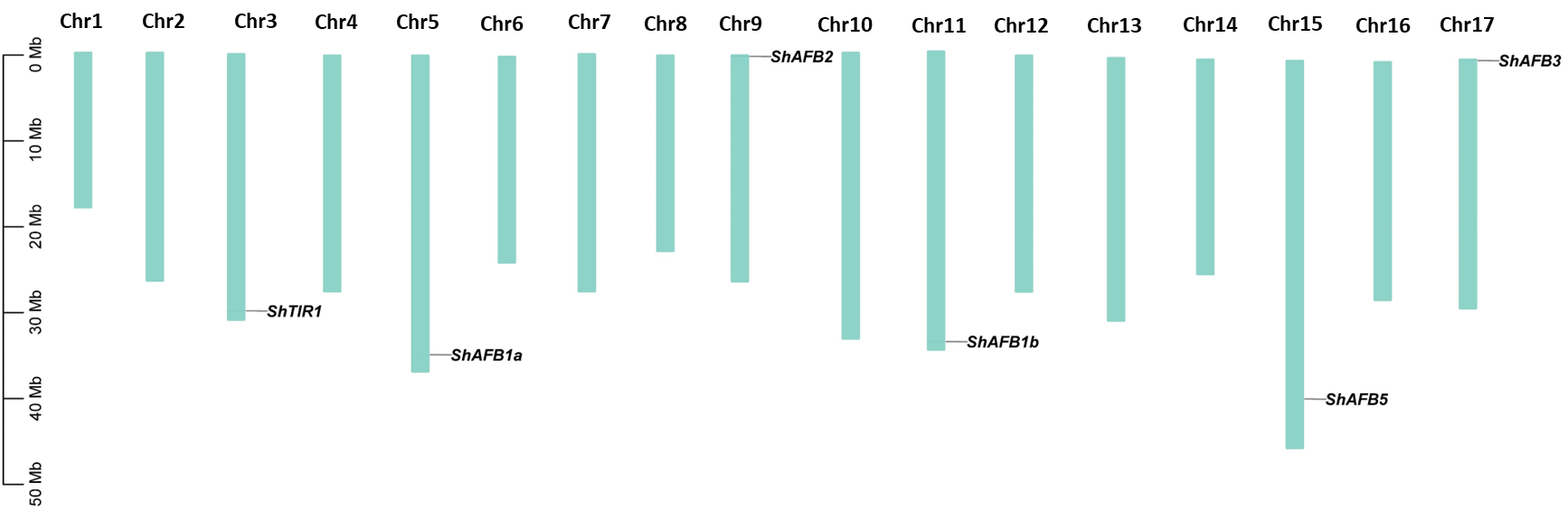


**Supplementary Figure 2:** *ShTIR1/AFBs* distributed on Shanxi Duli chromosomes.


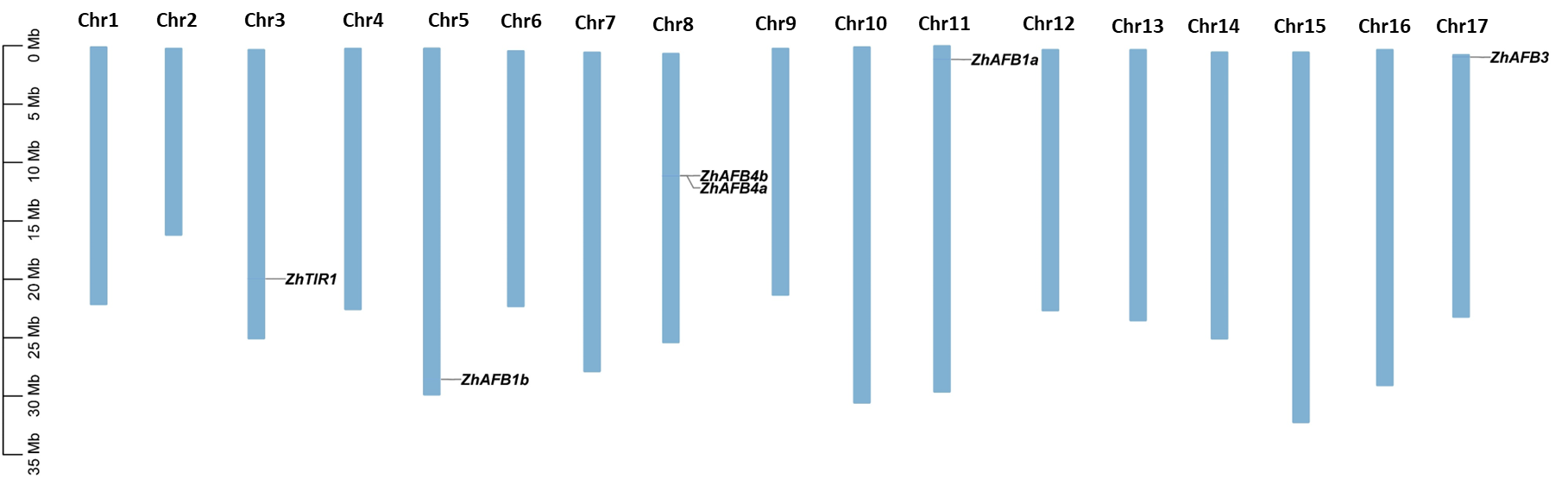


**Supplementary Figure 3:** *ZhTIR1/AFBs* distributed on Zhongai1 chromosomes.


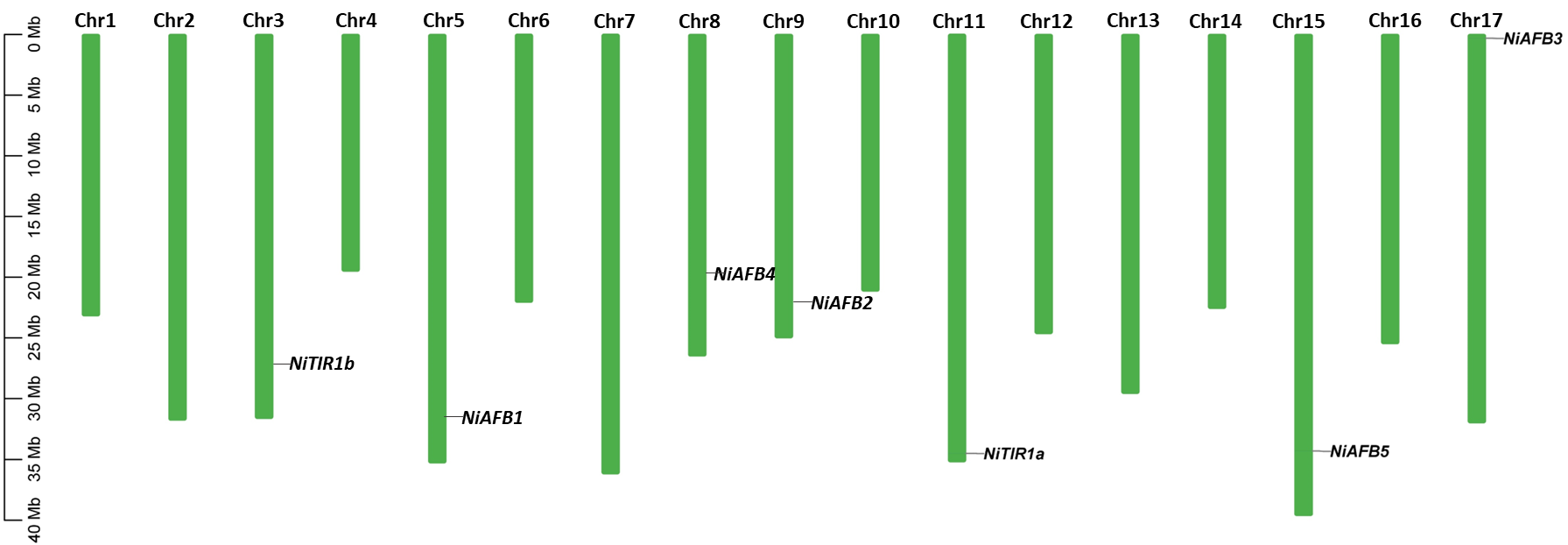


**Supplementary Figure 4:** *NiTIR1/AFBs* distributed on Nijisseiki chromosomes.


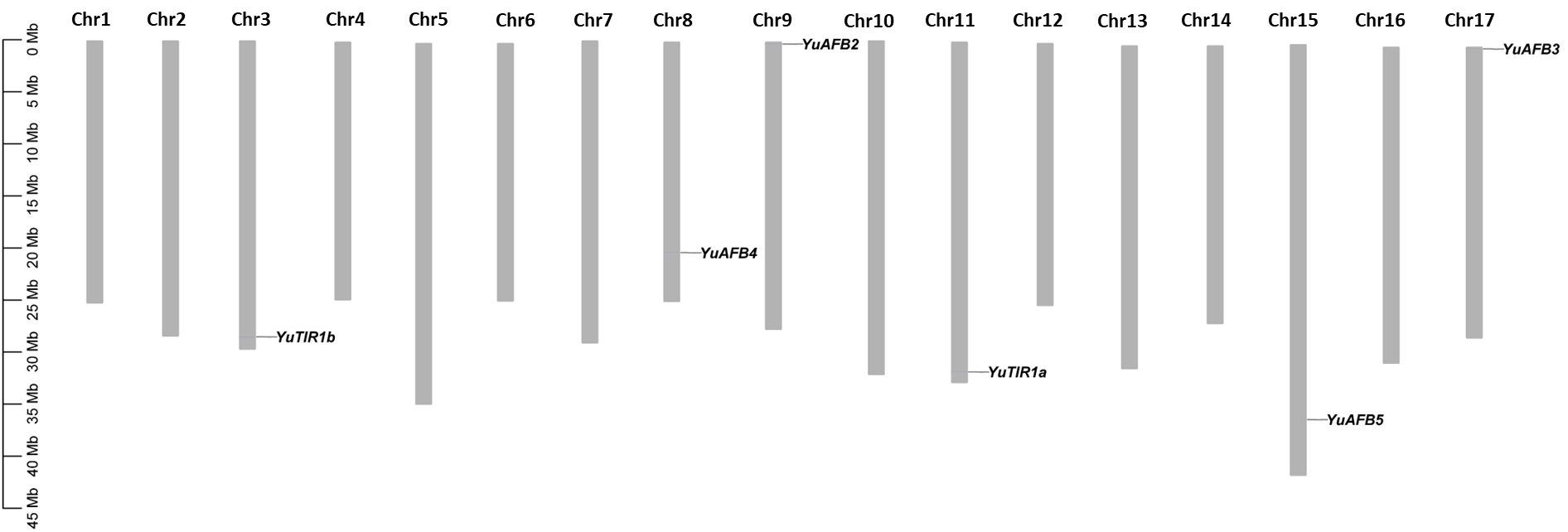


**Supplementary Figure 5:** *YuTIR1/AFBs* distributed on Yunhong No.1 chromosomes.


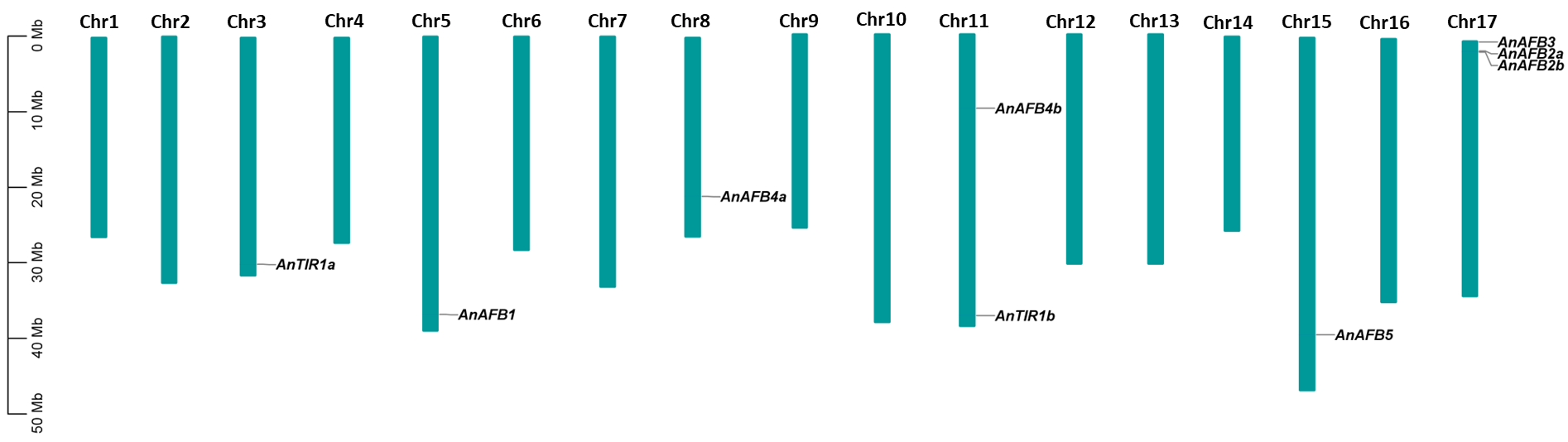


**Supplementary Figure 6:** *AnTIR1/AFBs* distributed on d’Anjou chromosomes.


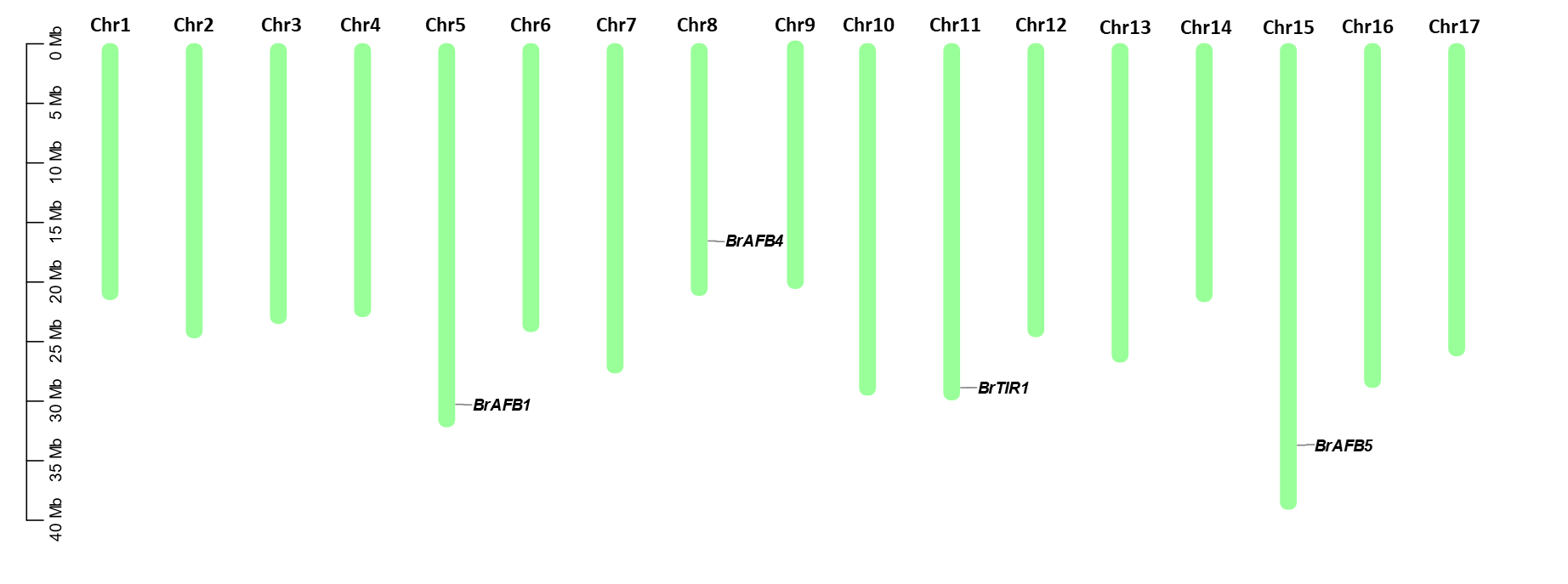


**Supplementary Figure 7:** *BrTIR1/AFBs* distributed on Bartlett chromosomes.

**Supplementary Table 1:** Identified *cis*-elements in Pyrus *TIR1/AFB* genes.

| **Gene** | **GATA-motif** | **G-box** | **GT1-motif** | **Box 4** | **TCA-element** | **ABRE** | **P-box** | **CGTCA-motif** | **TGA-element** | **LTR** | **WUN-motif** | **GC-motif** | **MBS** | **TC-rich repeats** | **HD-Zip 1** | **CAT-box** | **O2-site** | **CircAdian** | **MBSI** |
| --- | --- | --- | --- | --- | --- | --- | --- | --- | --- | --- | --- | --- | --- | --- | --- | --- | --- | --- | --- |
| *CuAFB5* | 3 | 2 | 1 | 0 | 4 | 0 | 2 | 1 | 3 | 0 | 0 | 1 | 0 | 1 | 2 | 0 | 3 | 1 | 0 |
| *CuTIR1* | 4 | 2 | 2 | 1 | 0 | 2 | 1 | 2 | 6 | 1 | 0 | 11 | 1 | 1 | 1 | 0 | 0 | 11 | 2 |
| *CuAFB1a* | 2 | 5 | 5 | 1 | 0 | 0 | 0 | 1 | 4 | 1 | 0 | 0 | 0 | 0 | 1 | 0 | 0 | 0 | 0 |
| *CuAFB3* | 1 | 1 | 4 | 2 | 0 | 0 | 0 | 0 | 8 | 0 | 0 | 0 | 0 | 0 | 2 | 2 | 0 | 0 | 0 |
| *CuAFB4* | 3 | 3 | 3 | 1 | 0 | 1 | 0 | 2 | 6 | 0 | 0 | 0 | 0 | 1 | 1 | 0 | 0 | 0 | 1 |
| *CuAFB1b* | 6 | 0 | 0 | 1 | 1 | 0 | 0 | 1 | 1 | 0 | 0 | 1 | 0 | 1 | 1 | 0 | 0 | 1 | 0 |
| *CuAFB2* | 8 | 3 | 2 | 0 | 0 | 0 | 0 | 2 | 4 | 1 | 0 | 0 | 0 | 0 | 0 | 0 | 0 | 0 | 0 |
| *ShTIR1* | 9 | 2 | 2 | 0 | 0 | 1 | 0 | 2 | 6 | 0 | 0 | 0 | 0 | 0 | 0 | 0 | 1 | 0 | 1 |
| *ShAFB2* | 1 | 0 | 1 | 1 | 1 | 1 | 2 | 2 | 6 | 1 | 1 | 0 | 0 | 0 | 1 | 0 | 0 | 0 | 1 |
| *ShAFB3* | 5 | 3 | 3 | 0 | 2 | 0 | 3 | 0 | 0 | 0 | 0 | 0 | 1 | 0 | 1 | 0 | 1 | 0 | 1 |
| *ShAFB5* | 3 | 0 | 5 | 2 | 0 | 0 | 0 | 2 | 3 | 0 | 1 | 1 | 0 | 0 | 1 | 0 | 0 | 1 | 2 |
| *ShAFB1a* | 0 | 1 | 1 | 3 | 0 | 1 | 1 | 3 | 2 | 1 | 1 | 0 | 0 | 0 | 3 | 0 | 0 | 0 | 1 |
| *ShAFB1b* | 2 | 1 | 2 | 1 | 1 | 1 | 0 | 0 | 1 | 0 | 0 | 0 | 0 | 0 | 1 | 0 | 1 | 0 | 1 |
| *ZhAFB1a* | 5 | 4 | 2 | 2 | 0 | 0 | 1 | 3 | 3 | 0 | 0 | 0 | 0 | 0 | 2 | 0 | 0 | 0 | 0 |
| *ZhAFB3* | 1 | 4 | 2 | 2 | 1 | 2 | 0 | 2 | 6 | 0 | 0 | 0 | 0 | 1 | 2 | 0 | 0 | 0 | 2 |
| *ZhAFB4a* | 3 | 0 | 2 | 0 | 1 | 0 | 0 | 0 | 8 | 0 | 0 | 0 | 0 | 0 | 0 | 0 | 1 | 0 | 0 |
| *ZhAFB4b* | 5 | 0 | 1 | 1 | 0 | 0 | 0 | 3 | 9 | 1 | 1 | 0 | 0 | 0 | 1 | 0 | 1 | 0 | 0 |
| *ZhTIR1* | 3 | 1 | 2 | 0 | 2 | 2 | 0 | 0 | 1 | 0 | 0 | 1 | 0 | 0 | 0 | 0 | 0 | 1 | 2 |
| *ZhAFB1b* | 6 | 1 | 6 | 1 | 0 | 1 | 0 | 5 | 3 | 1 | 0 | 0 | 0 | 0 | 0 | 0 | 1 | 0 | 1 |
| *NiAFB5* | 6 | 0 | 0 | 1 | 0 | 2 | 0 | 1 | 4 | 1 | 0 | 1 | 0 | 0 | 1 | 0 | 0 | 1 | 0 |
| *NiAFB4* | 3 | 2 | 0 | 0 | 0 | 0 | 0 | 4 | 0 | 0 | 0 | 0 | 0 | 0 | 0 | 0 | 0 | 0 | 0 |
| *NiTIR1a* | 6 | 0 | 2 | 0 | 1 | 1 | 0 | 4 | 2 | 0 | 0 | 1 | 0 | 0 | 0 | 0 | 0 | 1 | 1 |
| *NiAFB1* | 9 | 0 | 2 | 4 | 1 | 0 | 0 | 0 | 5 | 0 | 0 | 0 | 0 | 0 | 4 | 1 | 0 | 0 | 0 |
| *NiAFB3* | 3 | 0 | 1 | 0 | 0 | 0 | 0 | 0 | 1 | 1 | 0 | 0 | 1 | 0 | 0 | 0 | 0 | 0 | 0 |
| *NiAFB2* | 4 | 0 | 2 | 1 | 1 | 0 | 1 | 1 | 3 | 0 | 0 | 0 | 0 | 0 | 1 | 0 | 0 | 0 | 0 |
| *NiTIR1b* | 9 | 0 | 2 | 1 | 0 | 0 | 0 | 1 | 0 | 0 | 1 | 0 | 0 | 0 | 1 | 0 | 2 | 0 | 0 |
| *YuAFB2* | 9 | 0 | 0 | 0 | 0 | 1 | 0 | 0 | 0 | 1 | 0 | 0 | 0 | 0 | 0 | 0 | 1 | 0 | 1 |
| *YuTIR1a* | 2 | 0 | 2 | 0 | 0 | 1 | 0 | 2 | 0 | 0 | 0 | 1 | 0 | 0 | 0 | 0 | 0 | 1 | 1 |
| *YuAFB4* | 3 | 0 | 2 | 0 | 0 | 1 | 0 | 0 | 0 | 0 | 0 | 1 | 0 | 0 | 0 | 0 | 0 | 1 | 1 |
| *YuAFB5* | 3 | 2 | 1 | 0 | 0 | 0 | 1 | 0 | 0 | 0 | 0 | 2 | 0 | 0 | 0 | 1 | 0 | 2 | 0 |
| *YuTIR1b* | 7 | 0 | 0 | 2 | 0 | 0 | 0 | 0 | 0 | 0 | 0 | 0 | 0 | 0 | 2 | 0 | 0 | 0 | 0 |
| *YuAFB3* | 2 | 2 | 4 | 1 | 3 | 1 | 0 | 0 | 0 | 1 | 0 | 1 | 1 | 0 | 1 | 1 | 0 | 1 | 1 |
| *AnAFB3* | 11 | 2 | 1 | 1 | 0 | 0 | 0 | 0 | 0 | 0 | 0 | 2 | 0 | 1 | 1 | 0 | 0 | 2 | 0 |
| *AnAFB2a* | 6 | 0 | 2 | 0 | 0 | 1 | 1 | 0 | 0 | 0 | 1 | 0 | 0 | 0 | 0 | 0 | 0 | 0 | 1 |
| *AnTIR1a* | 0 | 0 | 1 | 2 | 0 | 1 | 2 | 0 | 0 | 1 | 1 | 0 | 0 | 0 | 2 | 0 | 0 | 0 | 1 |
| *AnTIR1b* | 7 | 0 | 9 | 0 | 0 | 0 | 1 | 0 | 0 | 0 | 1 | 0 | 0 | 0 | 0 | 0 | 0 | 0 | 0 |
| *AnAFB1* | 4 | 1 | 3 | 0 | 0 | 0 | 0 | 0 | 0 | 0 | 0 | 0 | 0 | 0 | 2 | 0 | 0 | 0 | 2 |
| *AnAFB4b* | 5 | 0 | 4 | 0 | 0 | 0 | 0 | 0 | 0 | 0 | 0 | 0 | 0 | 0 | 0 | 0 | 0 | 0 | 0 |
| *AnAFB5* | 0 | 0 | 9 | 0 | 0 | 0 | 0 | 0 | 0 | 0 | 0 | 0 | 0 | 0 | 1 | 0 | 1 | 0 | 0 |
| *AnAFB4a* | 1 | 0 | 9 | 0 | 0 | 0 | 0 | 0 | 0 | 0 | 0 | 0 | 0 | 0 | 0 | 0 | 2 | 0 | 0 |
| *AnAFB2b* | 3 | 4 | 5 | 0 | 0 | 0 | 0 | 0 | 0 | 0 | 0 | 0 | 0 | 0 | 0 | 0 | 0 | 1 | 1 |
| *BrAFB4* | 1 | 0 | 3 | 0 | 0 | 0 | 0 | 0 | 0 | 0 | 0 | 0 | 0 | 0 | 2 | 0 | 0 | 0 | 0 |
| *BrAFB2* | 11 | 0 | 6 | 0 | 0 | 0 | 0 | 0 | 0 | 0 | 0 | 0 | 0 | 0 | 1 | 0 | 0 | 0 | 0 |
| *BrAFB5* | 2 | 2 | 6 | 0 | 0 | 0 | 0 | 0 | 0 | 0 | 0 | 0 | 0 | 0 | 2 | 0 | 0 | 0 | 1 |
| *BrAFB1* | 1 | 0 | 9 | 0 | 0 | 0 | 0 | 0 | 0 | 0 | 0 | 0 | 0 | 0 | 0 | 0 | 2 | 0 | 0 |
| *BrTIR1* | 9 | 2 | 2 | 0 | 0 | 1 | 0 | 2 | 6 | 0 | 0 | 0 | 0 | 0 | 0 | 0 | 1 | 0 | 1 |
| *DaAFB3a* | 6 | 0 | 0 | 1 | 0 | 2 | 0 | 1 | 4 | 1 | 0 | 1 | 0 | 0 | 1 | 0 | 0 | 1 | 0 |
| *DaAFB1* | 1 | 0 | 9 | 0 | 0 | 0 | 0 | 0 | 0 | 0 | 0 | 0 | 0 | 0 | 0 | 0 | 2 | 0 | 0 |
| *DaAFB5* | 6 | 0 | 0 | 1 | 0 | 2 | 0 | 1 | 4 | 1 | 0 | 1 | 0 | 0 | 1 | 0 | 0 | 1 | 0 |
| *DaAFB3b* | 2 | 2 | 4 | 1 | 3 | 1 | 0 | 0 | 0 | 1 | 0 | 1 | 1 | 0 | 1 | 1 | 0 | 1 | 1 |

**Supplementary Table 2:** Gene ids, their GO enrichment classes, and description of predicted GO terms.

| **Gene ID** | **GO Class** | **GO terms** |
| --- | --- | --- |
| *CuAFB5* | BP | SCF-dependent proteasomal ubiquitin-dependent protein catabolic process |
| *CuAFB5* | BP | auxin-activated signaling pathway |
| *CuTIR1* | BP | auxin-activated signaling pathway |
| *CuTIR1* | BP | SCF-dependent proteasomal ubiquitin-dependent protein catabolic process |
| *CuAFB1a* | BP | auxin-activated signaling pathway |
| *CuAFB1a* | BP | SCF-dependent proteasomal ubiquitin-dependent protein catabolic process |
| *CuAFB3* | BP | auxin-activated signaling pathway |
| *CuAFB3* | BP | SCF-dependent proteasomal ubiquitin-dependent protein catabolic process |
| *CuAFB3* | BP | cellular response to nitrate |
| *CuAFB3* | BP | pollen maturation |
| *CuAFB3* | BP | primary root development |
| *CuAFB3* | BP | lateral root development |
| *CuAFB3* | BP | stamen development |
| *CuAFB4* | BP | SCF-dependent proteasomal ubiquitin-dependent protein catabolic process |
| *CuAFB4* | BP | auxin-activated signaling pathway |
| *CuAFB1b* | BP | auxin-activated signaling pathway |
| *CuAFB1b* | BP | SCF-dependent proteasomal ubiquitin-dependent protein catabolic process |
| *CuAFB2* | BP | auxin-activated signaling pathway |
| *CuAFB2* | BP | SCF-dependent proteasomal ubiquitin-dependent protein catabolic process |
| *ShTIR1* | BP | auxin-activated signaling pathway |
| *ShTIR1* | BP | SCF-dependent proteasomal ubiquitin-dependent protein catabolic process |
| *ShAFB2* | BP | auxin-activated signaling pathway |
| *ShAFB2* | BP | SCF-dependent proteasomal ubiquitin-dependent protein catabolic process |
| *ShAFB3* | BP | auxin-activated signaling pathway |
| *ShAFB3* | BP | SCF-dependent proteasomal ubiquitin-dependent protein catabolic process |
| *ShAFB3* | BP | cellular response to nitrate |
| *ShAFB3* | BP | pollen maturation |
| *ShAFB3* | BP | primary root development |
| *ShAFB3* | BP | lateral root development |
| *ShAFB3* | BP | stamen development |
| *ShAFB5* | BP | SCF-dependent proteasomal ubiquitin-dependent protein catabolic process |
| *ShAFB5* | BP | auxin-activated signaling pathway |
| *ShAFB1a* | BP | auxin-activated signaling pathway |
| *ShAFB1a* | BP | SCF-dependent proteasomal ubiquitin-dependent protein catabolic process |
| *ShAFB1b* | BP | auxin-activated signaling pathway |
| *ShAFB1b* | BP | SCF-dependent proteasomal ubiquitin-dependent protein catabolic process |
| *ZhAFB1a* | BP | auxin-activated signaling pathway |
| *ZhAFB1a* | BP | SCF-dependent proteasomal ubiquitin-dependent protein catabolic process |
| *ZhAFB1a* | BP | pollen maturation |
| *ZhAFB1a* | BP | lateral root formation |
| *ZhAFB1a* | BP | stamen development |
| *ZhAFB1a* | BP | cellular response to phosphate starvation |
| *ZhAFB1a* | BP | protein ubiquitination |
| *ZhAFB1a* | BP | defense response |
| *ZhAFB1a* | BP | ethylene-activated signaling pathway |
| *ZhAFB1a* | BP | response to other organism |
| *ZhAFB1a* | BP | cell cycle |
| *ZhAFB3* | BP | auxin-activated signaling pathway |
| *ZhAFB3* | BP | SCF-dependent proteasomal ubiquitin-dependent protein catabolic process |
| *ZhAFB4a* | BP | SCF-dependent proteasomal ubiquitin-dependent protein catabolic process |
| *ZhAFB4a* | BP | auxin-activated signaling pathway |
| *ZhAFB4b* | BP | SCF-dependent proteasomal ubiquitin-dependent protein catabolic process |
| *ZhAFB4b* | BP | auxin-activated signaling pathway |
| *ZhTIR1* | BP | auxin-activated signaling pathway |
| *ZhTIR1* | BP | SCF-dependent proteasomal ubiquitin-dependent protein catabolic process |
| *ZhAFB1b* | BP | auxin-activated signaling pathway |
| *ZhAFB1b* | BP | SCF-dependent proteasomal ubiquitin-dependent protein catabolic process |
| *NiAFB5* | BP | SCF-dependent proteasomal ubiquitin-dependent protein catabolic process |
| *NiAFB5* | BP | auxin-activated signaling pathway |
| *NiAFB4* | BP | SCF-dependent proteasomal ubiquitin-dependent protein catabolic process |
| *NiAFB4* | BP | auxin-activated signaling pathway |
| *NiTIR1a* | BP | auxin-activated signaling pathway |
| *NiTIR1a* | BP | SCF-dependent proteasomal ubiquitin-dependent protein catabolic process |
| *NiAFB1* | BP | auxin-activated signaling pathway |
| *NiAFB1* | BP | SCF-dependent proteasomal ubiquitin-dependent protein catabolic process |
| *NiAFB3* | BP | auxin-activated signaling pathway |
| *NiAFB3* | BP | SCF-dependent proteasomal ubiquitin-dependent protein catabolic process |
| *NiAFB3* | BP | cellular response to nitrate |
| *NiAFB3* | BP | pollen maturation |
| *NiAFB3* | BP | primary root development |
| *NiAFB3* | BP | lateral root development |
| *NiAFB3* | BP | stamen development |
| *NiAFB2* | BP | auxin-activated signaling pathway |
| *NiAFB2* | BP | SCF-dependent proteasomal ubiquitin-dependent protein catabolic process |
| *NiTIR1b* | BP | auxin-activated signaling pathway |
| *NiTIR1b* | BP | SCF-dependent proteasomal ubiquitin-dependent protein catabolic process |
| *YuAFB2* | BP | auxin-activated signaling pathway |
| *YuAFB2* | BP | SCF-dependent proteasomal ubiquitin-dependent protein catabolic process |
| *YuTIR1a* | BP | auxin-activated signaling pathway |
| *YuTIR1a* | BP | SCF-dependent proteasomal ubiquitin-dependent protein catabolic process |
| *YuAFB4* | BP | SCF-dependent proteasomal ubiquitin-dependent protein catabolic process |
| *YuAFB4* | BP | auxin-activated signaling pathway |
| *YuAFB5* | BP | SCF-dependent proteasomal ubiquitin-dependent protein catabolic process |
| *YuAFB5* | BP | auxin-activated signaling pathway |
| *YuTIR1b* | BP | auxin-activated signaling pathway |
| *YuTIR1b* | BP | SCF-dependent proteasomal ubiquitin-dependent protein catabolic process |
| *YuAFB3* | BP | auxin-activated signaling pathway |
| *YuAFB3* | BP | SCF-dependent proteasomal ubiquitin-dependent protein catabolic process |
| *YuAFB3* | BP | cellular response to nitrate |
| *YuAFB3* | BP | pollen maturation |
| *YuAFB3* | BP | primary root development |
| *YuAFB3* | BP | lateral root development |
| *YuAFB3* | BP | stamen development |
| *AnAFB3* | BP | auxin-activated signaling pathway |
| *AnAFB3* | BP | SCF-dependent proteasomal ubiquitin-dependent protein catabolic process |
| *AnAFB2a* | BP | auxin-activated signaling pathway |
| *AnAFB2a* | BP | SCF-dependent proteasomal ubiquitin-dependent protein catabolic process |
| *AnTIR1a* | BP | auxin-activated signaling pathway |
| *AnTIR1a* | BP | SCF-dependent proteasomal ubiquitin-dependent protein catabolic process |
| *AnTIR1b* | BP | auxin-activated signaling pathway |
| *AnTIR1b* | BP | SCF-dependent proteasomal ubiquitin-dependent protein catabolic process |
| *AnTIR1b* | BP | pollen maturation |
| *AnTIR1b* | BP | lateral root formation |
| *AnTIR1b* | BP | stamen development |
| *AnTIR1b* | BP | cellular response to phosphate starvation |
| *AnTIR1b* | BP | protein ubiquitination |
| *AnTIR1b* | BP | ethylene-activated signaling pathway |
| *AnTIR1b* | BP | defense response |
| *AnTIR1b* | BP | cell cycle |
| *AnAFB1* | BP | auxin-activated signaling pathway |
| *AnAFB1* | BP | SCF-dependent proteasomal ubiquitin-dependent protein catabolic process |
| *AnAFB4b* | BP | SCF-dependent proteasomal ubiquitin-dependent protein catabolic process |
| *AnAFB4b* | BP | auxin-activated signaling pathway |
| *AnAFB5* | BP | SCF-dependent proteasomal ubiquitin-dependent protein catabolic process |
| *AnAFB5* | BP | auxin-activated signaling pathway |
| *AnAFB4a* | BP | SCF-dependent proteasomal ubiquitin-dependent protein catabolic process |
| *AnAFB4a* | BP | auxin-activated signaling pathway |
| *AnAFB2b* | BP | auxin-activated signaling pathway |
| *AnAFB2b* | BP | SCF-dependent proteasomal ubiquitin-dependent protein catabolic process |
| *BrAFB4* | BP | SCF-dependent proteasomal ubiquitin-dependent protein catabolic process |
| *BrAFB4* | BP | auxin-activated signaling pathway |
| *BrAFB2* | BP | auxin-activated signaling pathway |
| *BrAFB2* | BP | SCF-dependent proteasomal ubiquitin-dependent protein catabolic process |
| *BrAFB5* | BP | SCF-dependent proteasomal ubiquitin-dependent protein catabolic process |
| *BrAFB5* | BP | auxin-activated signaling pathway |
| *BrAFB1* | BP | auxin-activated signaling pathway |
| *BrAFB1* | BP | SCF-dependent proteasomal ubiquitin-dependent protein catabolic process |
| *BrTIR1* | BP | auxin-activated signaling pathway |
| *BrTIR1* | BP | defense response to other organism |
| *DaAFB3a* | BP | auxin-activated signaling pathway |
| *DaAFB3a* | BP | SCF-dependent proteasomal ubiquitin-dependent protein catabolic process |
| *DaAFB1* | BP | auxin-activated signaling pathway |
| *DaAFB1* | BP | SCF-dependent proteasomal ubiquitin-dependent protein catabolic process |
| *DaAFB5* | BP | SCF-dependent proteasomal ubiquitin-dependent protein catabolic process |
| *DaAFB5* | BP | auxin-activated signaling pathway |
| *DaAFB3b* | BP | auxin-activated signaling pathway |
| *DaAFB3b* | BP | SCF-dependent proteasomal ubiquitin-dependent protein catabolic process |
| *DaAFB3b* | BP | cellular response to nitrate |
| *DaAFB3b* | BP | pollen maturation |
| *DaAFB3b* | BP | primary root development |
| *DaAFB3b* | BP | lateral root development |
| *DaAFB3b* | BP | stamen development |
| *CuAFB5* | CC | SCF ubiquitin ligase complex |
| *CuTIR1* | CC | SCF ubiquitin ligase complex |
| *CuAFB1a* | CC | SCF ubiquitin ligase complex |
| *CuAFB3* | CC | SCF ubiquitin ligase complex |
| *CuAFB4* | CC | SCF ubiquitin ligase complex |
| *CuAFB1b* | CC | SCF ubiquitin ligase complex |
| *CuAFB2* | CC | SCF ubiquitin ligase complex |
| *ShTIR1* | CC | SCF ubiquitin ligase complex |
| *ShAFB2* | CC | SCF ubiquitin ligase complex |
| *ShAFB3* | CC | SCF ubiquitin ligase complex |
| *ShAFB5* | CC | SCF ubiquitin ligase complex |
| *ShAFB1a* | CC | SCF ubiquitin ligase complex |
| *ShAFB1b* | CC | SCF ubiquitin ligase complex |
| *ZhAFB1a* | CC | SCF ubiquitin ligase complex |
| *ZhAFB1a* | CC | nucleus |
| *ZhAFB3* | CC | SCF ubiquitin ligase complex |
| *ZhAFB4a* | CC | SCF ubiquitin ligase complex |
| *ZhAFB4b* | CC | SCF ubiquitin ligase complex |
| *ZhTIR1* | CC | SCF ubiquitin ligase complex |
| *ZhAFB1b* | CC | SCF ubiquitin ligase complex |
| *NiAFB5* | CC | SCF ubiquitin ligase complex |
| *NiAFB4* | CC | SCF ubiquitin ligase complex |
| *NiTIR1a* | CC | SCF ubiquitin ligase complex |
| *NiAFB1* | CC | SCF ubiquitin ligase complex |
| *NiAFB3* | CC | SCF ubiquitin ligase complex |
| *NiAFB2* | CC | SCF ubiquitin ligase complex |
| *NiTIR1b* | CC | SCF ubiquitin ligase complex |
| *YuAFB2* | CC | SCF ubiquitin ligase complex |
| *YuTIR1a* | CC | SCF ubiquitin ligase complex |
| *YuAFB4* | CC | SCF ubiquitin ligase complex |
| *YuAFB5* | CC | SCF ubiquitin ligase complex |
| *YuTIR1b* | CC | SCF ubiquitin ligase complex |
| *YuAFB3* | CC | SCF ubiquitin ligase complex |
| *AnAFB3* | CC | SCF ubiquitin ligase complex |
| *AnAFB2a* | CC | SCF ubiquitin ligase complex |
| *AnTIR1a* | CC | SCF ubiquitin ligase complex |
| *AnTIR1b* | CC | SCF ubiquitin ligase complex |
| *AnTIR1b* | CC | nucleus |
| *AnAFB1* | CC | SCF ubiquitin ligase complex |
| *AnAFB4b* | CC | SCF ubiquitin ligase complex |
| *AnAFB5* | CC | SCF ubiquitin ligase complex |
| *AnAFB4a* | CC | SCF ubiquitin ligase complex |
| *AnAFB2b* | CC | SCF ubiquitin ligase complex |
| *BrAFB4* | CC | SCF ubiquitin ligase complex |
| *BrAFB2* | CC | SCF ubiquitin ligase complex |
| *BrAFB5* | CC | SCF ubiquitin ligase complex |
| *BrAFB1* | CC | SCF ubiquitin ligase complex |
| *BrTIR1* | CC | cytoplasm |
| *DaAFB3a* | CC | SCF ubiquitin ligase complex |
| *DaAFB1* | CC | SCF ubiquitin ligase complex |
| *DaAFB5* | CC | SCF ubiquitin ligase complex |
| *DaAFB3b* | CC | SCF ubiquitin ligase complex |
| *CuAFB5* | MF | inositol hexakisphosphate binding |
| *CuTIR1* | MF | inositol hexakisphosphate binding |
| *CuAFB1a* | MF | inositol hexakisphosphate binding |
| *CuAFB3* | MF | auxin binding |
| *CuAFB4* | MF | inositol hexakisphosphate binding |
| *CuAFB1b* | MF | inositol hexakisphosphate binding |
| *ShTIR1* | MF | inositol hexakisphosphate binding |
| *ShAFB3* | MF | auxin binding |
| *ShAFB5* | MF | inositol hexakisphosphate binding |
| *ShAFB1a* | MF | inositol hexakisphosphate binding |
| *ShAFB1b* | MF | inositol hexakisphosphate binding |
| *ZhAFB1a* | MF | inositol hexakisphosphate binding |
| *ZhAFB1a* | MF | auxin receptor activity |
| *ZhAFB1a* | MF | auxin binding |
| *ZhAFB1a* | MF | ubiquitin-protein transferase activity |
| *ZhAFB1a* | MF | protein binding |
| *ZhAFB4a* | MF | inositol hexakisphosphate binding |
| *ZhAFB4b* | MF | inositol hexakisphosphate binding |
| *ZhTIR1* | MF | inositol hexakisphosphate binding |
| *ZhAFB1b* | MF | inositol hexakisphosphate binding |
| *NiAFB5* | MF | inositol hexakisphosphate binding |
| *NiAFB4* | MF | inositol hexakisphosphate binding |
| *NiTIR1a* | MF | inositol hexakisphosphate binding |
| *NiAFB1* | MF | inositol hexakisphosphate binding |
| *NiAFB3* | MF | auxin binding |
| *NiTIR1b* | MF | inositol hexakisphosphate binding |
| *YuTIR1a* | MF | inositol hexakisphosphate binding |
| *YuAFB4* | MF | inositol hexakisphosphate binding |
| *YuAFB5* | MF | inositol hexakisphosphate binding |
| *YuTIR1b* | MF | inositol hexakisphosphate binding |
| *YuAFB3* | MF | auxin binding |
| *AnTIR1a* | MF | inositol hexakisphosphate binding |
| *AnTIR1b* | MF | inositol hexakisphosphate binding |
| *AnTIR1b* | MF | auxin receptor activity |
| *AnTIR1b* | MF | auxin binding |
| *AnTIR1b* | MF | ubiquitin-protein transferase activity |
| *AnTIR1b* | MF | protein binding |
| *AnAFB1* | MF | inositol hexakisphosphate binding |
| *AnAFB4b* | MF | inositol hexakisphosphate binding |
| *AnAFB5* | MF | inositol hexakisphosphate binding |
| *AnAFB4a* | MF | inositol hexakisphosphate binding |
| *BrAFB4* | MF | inositol hexakisphosphate binding |
| *BrAFB5* | MF | inositol hexakisphosphate binding |
| *BrAFB1* | MF | inositol hexakisphosphate binding |
| *DaAFB1* | MF | inositol hexakisphosphate binding |
| *DaAFB5* | MF | inositol hexakisphosphate binding |
| *DaAFB3b* | MF | auxin binding |

**Supplementary Table 3:** Domains identified in Pyrus TIR1/AFB members.

| **TIR1/AFBs** | **CDD** | **Interpro** |
| --- | --- | --- |
| CuTIR1 | Transp_inhibit, F-box_5 superfamily | F-box-like domain superfamily, COI1, F-box, Leucine-rich repeat, cysteine-containing subtype, Transport inhibitor response 1 domain, Leucine-rich repeat domain superfamily, F-box domain |
| CuAFB1a | Transp_inhibit, F-box_5 superfamily | Transport inhibitor response 1 domain, Leucine-rich repeat, COI1, F-box, Leucine-rich repeat, cysteine-containing subtype, Leucine-rich repeat domain superfamily |
| CuAFB1b | Transp_inhibit, F-box_5 | Leucine-rich repeat domain superfamily, Leucine-rich repeat, cysteine-containing subtype, COI1, F-box, F-box domain, F-box-like domain superfamily, Transport inhibitor response 1 domain |
| CuAFB2 | Transp_inhibit, F-box_5 superfamily | F-box domain, Leucine-rich repeat domain superfamily, COI1, F-box, Transport inhibitor response 1 domain, Leucine-rich repeat, cysteine-containing subtype, Leucine-rich repeat |
| CuAFB3 | Transp_inhibit, F-box_5 superfamily | Leucine-rich repeat, Transport inhibitor response 1 domain, Leucine-rich repeat domain superfamily, COI1, F-box, F-box domain, Leucine-rich repeat, cysteine-containing subtype |
| CuAFB4 | Transp_inhibit, F-box_5 superfamily | Leucine-rich repeat, cysteine-containing subtype, Leucine-rich repeat domain superfamily, Transport inhibitor response 1 domain |
| CuAFB5 | Transp_inhibit, F-box_5 | COI1, F-box, Leucine-rich repeat domain superfamily, Transport inhibitor response 1 domain |
| ShTIR1 | Transp_inhibit, F-box_5 | Transport inhibitor response 1 domain, Leucine-rich repeat, cysteine-containing subtype, Leucine-rich repeat domain superfamily, F-box domain, COI1, F-box, F-box-like domain superfamily |
| ShAFB1a | Transp_inhibit, F-box_5 superfamily | Transport inhibitor response 1 domain, COI1, F-box, Leucine-rich repeat, cysteine-containing subtype, Leucine-rich repeat, Leucine-rich repeat domain superfamily |
| ShAFB1b | Transp_inhibit, F-box_5 | Transport inhibitor response 1 domain, Leucine-rich repeat domain superfamily, Leucine-rich repeat, cysteine-containing subtype, COI1, F-box, F-box domain, F-box-like domain superfamily |
| ShAFB2 | Transp_inhibit, F-box_5 superfamily | F-box domain, COI1, F-box, Transport inhibitor response 1 domain, Leucine-rich repeat domain superfamily, Leucine-rich repeat, cysteine-containing subtype, Leucine-rich repeat |
| ShAFB3 | Transp_inhibit, F-box_5 superfamily | Leucine-rich repeat domain superfamily, Leucine-rich repeat, Transport inhibitor response 1 domain, COI1, F-box, F-box domain, Leucine-rich repeat, cysteine-containing subtype |
| ShAFB5 | Transp_inhibit, F-box_5 | Leucine-rich repeat domain superfamily, COI1, F-box, Leucine-rich repeat, cysteine-containing subtype, Transport inhibitor response 1 domain |
| ZhTIR1 | Transp_inhibit, F-box_5 superfamily | COI1, F-box, F-box-like domain superfamily, F-box domain, Leucine-rich repeat, cysteine-containing subtype, Transport inhibitor response 1 domain, Leucine-rich repeat domain superfamily |
| ZhAFB1a | Transp_inhibit, F-box_5 | COI1, F-box, Transport inhibitor response 1 domain, Leucine-rich repeat, cysteine-containing subtype, F-box domain, F-box-like domain superfamily, Leucine-rich repeat domain superfamily |
| ZhAFB1b | Transp_inhibit, F-box_5 superfamily | Transport inhibitor response 1 domain, Leucine-rich repeat, Leucine-rich repeat, cysteine-containing subtype, Leucine-rich repeat domain superfamily, COI1, F-box |
| ZhAFB3 | Transp_inhibit, F-box_5 superfamily | Leucine-rich repeat, Leucine-rich repeat domain superfamily, F-box domain, Leucine-rich repeat, cysteine-containing subtype, Transport inhibitor response 1 domain, COI1, F-box |
| ZhAFB4a | Transp_inhibit, F-box_5 | Transport inhibitor response 1 domain, COI1, F-box, Leucine-rich repeat domain superfamily, F-box-like domain superfamily, Leucine-rich repeat, cysteine-containing subtype |
| ZhAFB4b | Transp_inhibit, F-box_5 | Leucine-rich repeat domain superfamily, Leucine-rich repeat, cysteine-containing subtype, Transport inhibitor response 1 domain, COI1, F-box |
| NiTIR1a | Transp_inhibit, F-box_5 | Leucine-rich repeat, cysteine-containing subtype, COI1, F-box, Leucine-rich repeat domain superfamily, F-box domain, F-box-like domain superfamily, Transport inhibitor response 1 domain |
| NiTIR1b | Transp_inhibit, F-box_5 superfamily | F-box-like domain superfamily, COI1, F-box, Leucine-rich repeat, cysteine-containing subtype, Transport inhibitor response 1 domain, Leucine-rich repeat domain superfamily, F-box domain |
| NiAFB1 | Transp_inhibit, F-box_5 superfamily | Leucine-rich repeat domain superfamily, COI1, F-box, Leucine-rich repeat, Leucine-rich repeat, cysteine-containing subtype, Transport inhibitor response 1 domain |
| NiAFB2 | Transp_inhibit, F-box_5 superfamily | F-box domain, Leucine-rich repeat domain superfamily, Transport inhibitor response 1 domain, COI1, F-box, Leucine-rich repeat, cysteine-containing subtype |
| NiAFB3 | Transp_inhibit, F-box_5 superfamily | Leucine-rich repeat, cysteine-containing subtype, Transport inhibitor response 1 domain, COI1, F-box, F-box domain, Leucine-rich repeat domain superfamily, Leucine-rich repeat |
| NiAFB4 | Transp_inhibit, F-box_5 | Transport inhibitor response 1 domain, Leucine-rich repeat domain superfamily, F-box-like domain superfamily, COI1, F-box, Leucine-rich repeat, cysteine-containing subtype |
| NiAFB5 | Transp_inhibit, F-box_5 | COI1, F-box, Leucine-rich repeat, cysteine-containing subtype, Transport inhibitor response 1 domain, Leucine-rich repeat domain superfamily |
| YuTIR1a | Transp_inhibit, F-box_5 | Transport inhibitor response 1 domain, Leucine-rich repeat domain superfamily, COI1, F-box, Leucine-rich repeat, cysteine-containing subtype |
| YuTIR1b | Transp_inhibit, F-box_5 superfamily | Leucine-rich repeat, cysteine-containing subtype, Transport inhibitor response 1 domain, COI1, F-box, F-box domain, Leucine-rich repeat domain superfamily, Leucine-rich repeat |
| YuAFB2 | Transp_inhibit, F-box_5 superfamily | Leucine-rich repeat, cysteine-containing subtype, Transport inhibitor response 1 domain, COI1, F-box, F-box domain, Leucine-rich repeat domain superfamily, Leucine-rich repeat |
| YuAFB3 | Transp_inhibit, F-box_5 superfamily | Leucine-rich repeat, cysteine-containing subtype, Transport inhibitor response 1 domain, COI1, F-box, F-box domain, Leucine-rich repeat domain superfamily, Leucine-rich repeat |
| YuAFB4 | Transp_inhibit, F-box_5 | COI1, F-box, Leucine-rich repeat, Leucine-rich repeat, cysteine-containing subtype, Leucine-rich repeat domain superfamily, Transport inhibitor response 1 domain |
| YuAFB5 | Transp_inhibit, F-box_5 | Transport inhibitor response 1 domain, Leucine-rich repeat domain superfamily, COI1, F-box, Leucine-rich repeat, cysteine-containing subtype |
| AnTIR1a | Transp_inhibit, F-box_5 superfamily | F-box-like domain superfamily, COI1, F-box, Leucine-rich repeat, cysteine-containing subtype, Transport inhibitor response 1 domain, Leucine-rich repeat domain superfamily, F-box domain |
| AnTIR1b | Transp_inhibit, F-box_5 | Leucine-rich repeat, cysteine-containing subtype, Transport inhibitor response 1 domain, COI1, F-box, F-box domain, Leucine-rich repeat domain superfamily, Leucine-rich repeat |
| AnAFB1 | Transp_inhibit, F-box_5 superfamily | COI1, F-box, Leucine-rich repeat, Leucine-rich repeat, cysteine-containing subtype, Leucine-rich repeat domain superfamily, Transport inhibitor response 1 domain |
| AnAFB2a | Transp_inhibit, F-box_5 | F-box domain, Leucine-rich repeat, Transport inhibitor response 1 domain, COI1, F-box, Leucine-rich repeat domain superfamily, Leucine-rich repeat, cysteine-containing subtype |
| AnAFB2b | Transp_inhibit, F-box_5 superfamily | F-box domain, Leucine-rich repeat domain superfamily, Leucine-rich repeat, Transport inhibitor response 1 domain, COI1, F-box, Leucine-rich repeat, cysteine-containing subtype |
| AnAFB3 | Transp_inhibit, F-box_5 superfamily | Transport inhibitor response 1 domain, COI1, F-box, Leucine-rich repeat domain superfamily, F-box domain, Leucine-rich repeat, cysteine-containing subtype, Leucine-rich repeat |
| AnAFB4a | Transp_inhibit, F-box_5 | Transport inhibitor response 1 domain, COI1, F-box, Leucine-rich repeat domain superfamily, F-box-like domain superfamily, Leucine-rich repeat, cysteine-containing subtype |
| AnAFB4b | Transp_inhibit, F-box_5 | Leucine-rich repeat, cysteine-containing subtype, Transport inhibitor response 1 domain, COI1, F-box, F-box domain, Leucine-rich repeat domain superfamily, Leucine-rich repeat |
| AnAFB5 | Transp_inhibit, F-box_5 | Transport inhibitor response 1 domain, Leucine-rich repeat, cysteine-containing subtype, COI1, F-box, Leucine-rich repeat domain superfamily |
| BrTIR1 | Transp_inhibit, F-box_5 | Transport inhibitor response 1 domain, Leucine-rich repeat domain superfamily, F-box-like domain superfamily, COI1, F-box, Leucine-rich repeat, cysteine-containing subtype |
| BrAFB1 | Transp_inhibit, F-box_5 superfamily | Transport inhibitor response 1 domain, Leucine-rich repeat domain superfamily, F-box-like domain superfamily, COI1, F-box, Leucine-rich repeat, cysteine-containing subtype |
| BrAFB2 | Transp_inhibit, F-box_5 superfamily | Leucine-rich repeat, cysteine-containing subtype, Transport inhibitor response 1 domain, COI1, F-box, F-box domain, Leucine-rich repeat domain superfamily, Leucine-rich repeat |
| BrAFB4 | Transp_inhibit, F-box_5 | Transport inhibitor response 1 domain, Leucine-rich repeat domain superfamily, F-box-like domain superfamily, COI1, F-box, Leucine-rich repeat, cysteine-containing subtype |
| BrAFB5 | Transp_inhibit, F-box_5 | Leucine-rich repeat, cysteine-containing subtype, Transport inhibitor response 1 domain, COI1, F-box, F-box domain, Leucine-rich repeat domain superfamily, Leucine-rich repeat |
| DaAFB1 | Transp_inhibit, F-box_5 superfamily | Transport inhibitor response 1 domain, Leucine-rich repeat domain superfamily, COI1, F-box, Leucine-rich repeat, cysteine-containing subtype |
| DaAFB3a | Transp_inhibit, F-box_5 superfamily | Leucine-rich repeat, cysteine-containing subtype, Transport inhibitor response 1 domain, COI1, F-box, F-box domain, Leucine-rich repeat domain superfamily, Leucine-rich repeat |
| DaAFB3b | Transp_inhibit, F-box_5 superfamily | Leucine-rich repeat, cysteine-containing subtype, Transport inhibitor response 1 domain, COI1, F-box, F-box domain, Leucine-rich repeat domain superfamily, Leucine-rich repeat |
| DaAFB5 | Transp_inhibit, F-box_5 | COI1, F-box, Transport inhibitor response 1 domain, Leucine-rich repeat, cysteine-containing subtype, Leucine-rich repeat domain superfamily |
